# N-Arylation of Protected and Unprotected 5-Bromo-2-aminobenzimidazole as Organic Material: Non-Linear Optical (NLO) Properties and Structural Feature Determination through Computational Approach

**DOI:** 10.3390/molecules26226920

**Published:** 2021-11-17

**Authors:** Mubeen Mumtaz, Nasir Rasool, Gulraiz Ahmad, Naveen Kosar, Umer Rashid

**Affiliations:** 1 Department of Chemistry, Government College University, Faisalabad 38000, Pakistan; advomubeen@ymail.com (M.M.); gulchemist35@gmail.com (G.A.); 2 Department of Chemistry, University of Management and Technology (UMT), C11, Johar Town, Lahore 54770, Pakistan; naveen.kosar@umt.edu.pk; 3 Department of Chemistry, COMSATS University Islamabad, Abbottabad Campus, Abbottabad 22060, Pakistan; 4 Institute of Nanoscience and Nanotechnology (ION2), Universiti Putra Malaysia (UPM), Serdang 43400, Selangor, Malaysia

**Keywords:** benzimidazole, N-arylation, optimization, FMOs analysis, non-linear optical properties

## Abstract

The interest in the NLO response of organic compounds is growing rapidly, due to the ease of synthesis, availability, and low loss. Here, in this study, Cu(II)-catalyzed selective N-arylation of 2-aminobenzimidazoles derivatives were achieved in the presence of different bases Et_3_N/TMEDA, solvents DCM/MeOH/H_2_O, and various aryl boronic acids under open atmospheric conditions. Two different copper-catalyzed pathways were selected for N-arylation in the presence of active nucleophilic sites, providing a unique tool for the preparation of NLO materials, C-NH (aryl) derivatives of 2-aminobenzimidazoles with protection and without protection of NH_2_ group. In addition to NMR analysis, all synthesized derivatives (**1a**–**1f** and **2a**–**2f**) of 5-bromo-2-aminobenzimidazole (**1**) were computed for their non-linear optical (NLO) properties and reactivity descriptor parameters. Frontier molecular orbital (FMO) analysis was performed to get information about the electronic properties and reactivity of synthesized compounds.

## 1. Introduction

There are 30 derivatives of 2-aminobenzimidazole registered in the world as drugs that exhibit diverse pharmacological activities, e.g., antiparasitic, antifungal, antiviral, and anti-allergic [1]. The 2-aminobenzimidazoles are interesting compounds with many biological effects, such as immunotropic, diuretic, antihistamine, and highly selective characteristics of p38aMaP inhibition [2,3,4]. Moreover, 2-aminobenzimidazole derivatives with antiviral activity against herpes simplex virus (HSV), human cyclomegalo virus (HCMV), and HIV have also been developed and patented [5,6,7]. The benzimidazole moiety is also a component of chemosensor receptors, which are utilized to recognize anions selectively and play an essential part in several biological activities [8]. The polyfunctionality of the cyclic guanidine moiety in the 2-aminobenzimidazole has made it a building block for the synthesis of a wide range of pharmacologically important benzimidazole derivatives [9,10]. Therefore, the selective N-arylation of amino-substituted N-heterocycles without protection and with protection of nucleophilic sites is important synthetically, as it gives an easy approach to a variety of different bio-actively potent N-arylated compounds.

Optimizing a catalytic pathway for substrates, where a couple of hetero atom sites are available for cross-coupling reactions, is an actual challenge for the organic chemist. The selective N-arylation of heteroaromatics, which has more than one nucleophilic site, could be precious because it quickly develops molecular complexity in target molecules with the fewest synthetic influences. However, a large number of transition metal-catalyzed protocols have developed as incredible tools for numerous types of regio/chemoselective N-(hetero) arylation reactions in organic synthesis [11]. Different Pd- and Cu-catalyzed arylations, such as Goldberg [12], Ullman [13], and Buchwald–Hartwig [14] procedures, were used for N-arylations of azoles. More recently, the copper-catalyzed Chan–Lam coupling method for arylation of N-nucleophile by using copper acetate and aryl boronic acids was introduced as the standard because of its mild reaction conditions [15,16]. The Chan–Lam coupling has been used for N-arylation of various amines, anilines, esters, imidazoles, and nitrogen-containing heterocycles [17].

These days computational chemistry [18] has emerged as a well-recognized partner of experimental chemistry. Computational chemistry is an extremely vast topic but herein we limited ourselves to density functional theory (DFT) [19]. Over the last 40 years, density functional theory (DFT) has become the most dominant and powerful tool in computational quantum chemistry for the modeling and simulation of chemical systems. Materials demonstrating great non-linear optical response play a key role in telecommunication, optical information processing, optical computing, etc. [20,21]. Nadeem and co-workers studied benzimidazole derivatives to tune the second-order nonlinear optical molecular switching by proton abstraction. Their results illustrate that substituted compounds have robustly large off–on NLO switching with a difference in *β*_o_ values of 7, 63, 85, and 75 times larger than their neutral counterparts, respectively [22]. Tayade and Sekar examined Benzimidazole-Thiazole based NLOphoric Styryl Dyes both experimentally and theoretically. The nonlinear response based on α, and β was increased after the substitution of acceptor groups [23]. Thakare et al. experimentally and theoretically analyzed the NLO response of BODIPY–benzimidazole conjugate. The results obtained from the DFT method are in good accordance with those produced from solvatochromic method. The values obtained for nonlinear absorption coefficient (*β*) and third-order susceptibility χ(3) are 7.45 × 10^12^ and 3.85 × 10^13^, respectively [24]. These reports shed light on the importance of substituted benzimidazole synthesis and their use in the field of optical and nonlinear optical materials using the DFT method.

In the present study, 2-aminobenzimidazole and its derivatives were synthesized via Chan–Lam cross-coupling reaction and analyzed theoretically for structural, spectroscopic [25], and NLO properties [26]. Density functional theory (DFT) calculations were used to investigate quantum chemical parameters, such as electron affinity (*E_A_*), ionization potential (*I*), electronic chemical potential (*μ*), electrophilicity index (*ω*), and chemical hardness (*η*) [27] of synthesized derivatives of 5-bromo-2-aminobenzimidazole. We hope that electron-donating and withdrawing groups substitution can enhance the NLO response of the newly designed bezimidazoles, and we will obtain large first-order hyperpolarizability for these compounds.

## 2. Results and Discussion

### 2.1. Chemistry

**Scheme 1:**
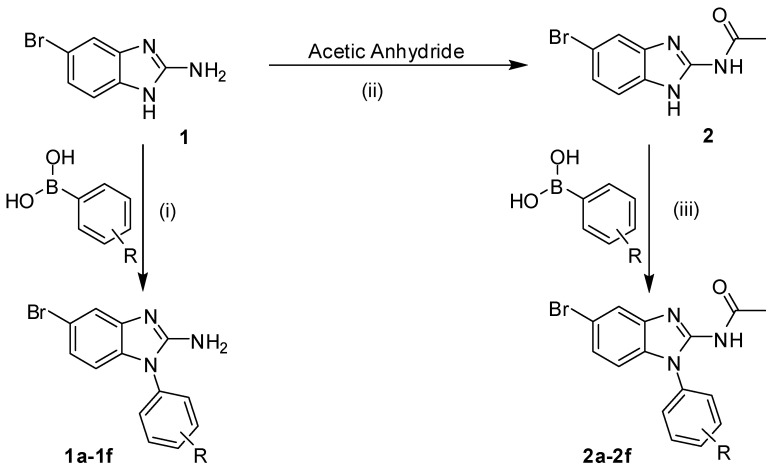
Synthesis of and 5-bromo-1*H*-benzo[*d*]imidazol-2-amine derivatives (**1a**–**1f**), *N*-(5-bromo-1*H*-benzo[*d*]imidazol-2-yl)acetamide (**2**), and *N*-(5-bromo-1*H*-benzo[*d*]imidazol-2-yl)acetamide derivatives (**2a**–**2f**). Reagents and conditions: (1) Cu(OAc)_2_ (0.94 mmol), **1** (0.94 mmol), Arylboronic acid (1.88 mmol), Et_3_N/TMEDA (1.88 mmol), MeOH/H_2_O (8:1), r.t., air, time of 2 h; (2) **1** (47 mmol), acetic anhydride (5 mL), temperature of 40 °C, time of 4 h; (3) Cu(OAc)_2_ (0.59 mmol), **2** (0.39 mmol), Arylboronic acid (0.47 mmol), Et_3_N/Py (0.59 mmol), DCM (10 mL), r.t., air, time of 24–72 h.

For the acquisition of optimized protocol for selective C-NH arylation of 5-bromo-2-amino benzimidazole, we initiated our examination by adopting two different protocols in which we used 5-bromo-2-aminobenzimidazole (**1**) and its derivative *N*-(5-bromo-1*H*-benzo[*d*]imidazol-2-yl)acetamide (**2**) with aryl boronic acids in presence of Cu(OAc)_2_ as a catalyst, Triethylamine (Et_3_N) or Tetramethylethylenediamine (TMEDA) as a base, and the solvent DCM and methanol. The reaction was carried out at normal temperature under an open-air environment (Scheme 1).

*N*-(5-bromo-1*H*-benzo[*d*]imidazol-2-yl)acetamide (**2**) was synthesized after selective protection of amino group of 5-bromo-2-aminobenzimidazole (**1**) by acetic anhydride at 40 °C through a previously reported method [28].

**Figure 1:**
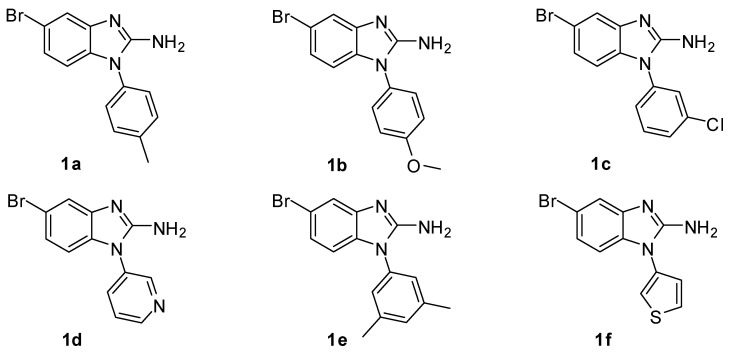
Synthesized 5-bromo-1*H*-benzo[*d*]imidazol-2-amine derivatives (**1a**–**1f**).

**Figure 2:**
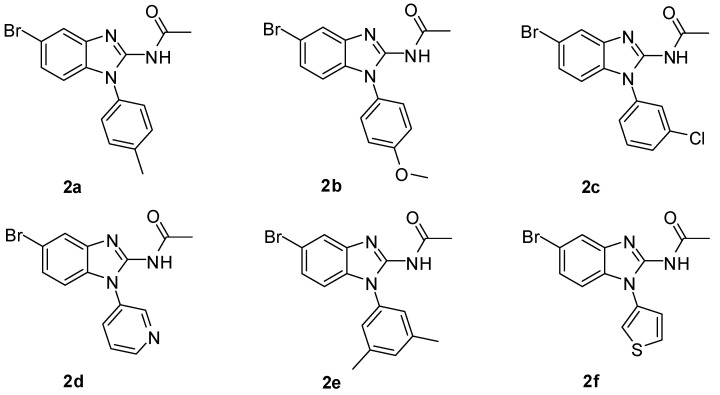
Synthesized *N*-(5-bromo-1*H*-benzo[*d*]imidazol-2-yl)acetamide derivatives (**2a**–**2f**).Different aryl/heteroaryl boronic acids were used in Suzuki Cross-coupling reactions under optimized conditions to get the desired derivatives of 5-bromo-2-aminobenzimidazole (**1**). The %age yield of various synthesized derivatives **1a**–**1f** and **2a**–**2f** are given in Table 1 and Table 2.

Generally, N-arylation of the heterocyclic compound with aryl boronic acid takes place under anhydrous conditions. In one protocol of direct N-arylation of 5-bromo-2-aminobenzimidazole (**1**) to produce its derivatives **1a**–**1f** (Figure 1), we used a TMEDA base and Cu(OAc)_2_·H_2_O catalyst with mixed parotic solvent (CH_3_OH/H_2_O 8:1). The desired products were obtained in good yield within only 2 h [29]. In contrast, in the second protocol for selective N-arylation of *N*-(5-bromo-1*H*-benzo[*d*]imidazol-2-yl)acetamide (**2**) to produce its derivatives **2a**–**2f** (Figure 2), we used anhydrous Cu(OAc)_2_ instead of Cu(OAc)_2_·H_2_O with aryl boronic acid in presence of dry DCM instead of CH_3_OH/H_2_O, Et_3_N, and 4 Å molecular sieves [15,30]. This method was not found efficient, as we failed to isolate the desired product in good yield, even after 24 h, and various tedious steps, such as protection of 5-bromo-2-aminobenzimidazole (**1**) involved in this method. It was noticed that, by the addition of TMEDA base and a small amount of water, the yield of desired products is enhanced significantly.

In the present study, it was observed that the base can significantly affect the Chan–Lam coupling. Et_3_N and pyridine are commonly used bases in Chan–Lam cross-coupling reactions. These bases contain amine additives which donate the electron pair to Cu(II) and allow it to oxidize Cu(III) intermediate and also capture the acetic acid which produces during the transmetallation step. For selective N-arylation of 5-bromo-2-aminobenzimidazole (**1**) without protection, we used TMEDA and Et_3_N as bases and obtained products in good yield with TMEDA base. It is observed that TMEDA act as a bidentate ligand and forms a more stable complex with copper which enhances its reactivity toward the NH group of imidazole ring as compared to Et_3_N for its selective N-arylation [31,32].

### 2.2. Density Functional Theory (DFT) Studies

**Figure 3:**
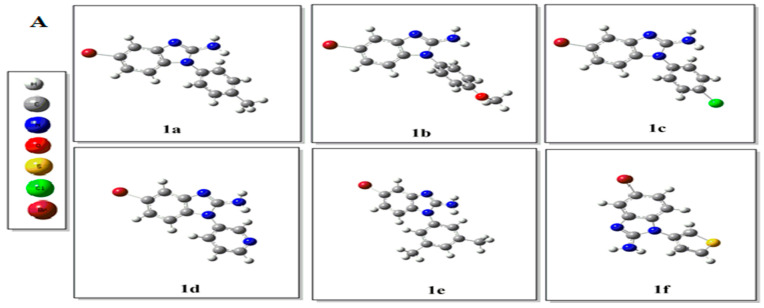

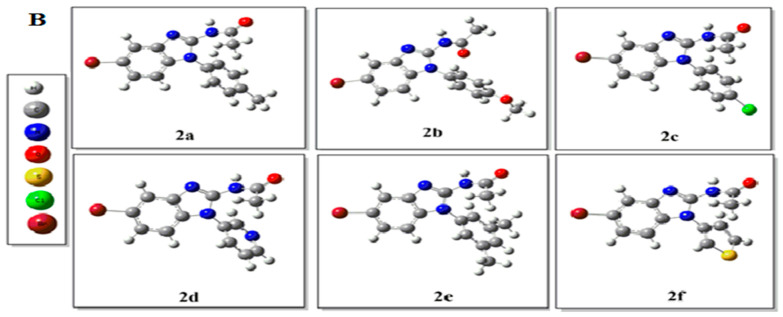
Optimized structures of (**A**) **1a**–**1f**; (**B**) **2a**–**2f** at B3LYP/6-31+G(d,p) level of theory.

All synthesized compounds (**1a**–**1f** and **2a**–**2f**) were computationally studied to investigate the structural–properties relationships. First of all, compounds were optimized at B3LYP functional of DFT by using GAUSSIAN 09 software [33]. For molecular orbitals description, Pople’6-31+G(d,p) [34] was used (Figure 3A,B). Frequency analysis were completed at the same level of theory for further confirmation of these structures as true minima energy structures on potential energy surfaces. A frontier molecular orbitals (FMOs) analysis and reactivity descriptor parameters were calculated at B3LYP/6-31+G(d,p) method [35]. Nonlinear optical (NLO) properties along with polarizabilities and first hyperpolarizability parameters were evaluated using CAM-B3LYP [36], LC-BLYP [37], and ωB97XD [38] density functionals with 6-31+G(d,p) basis set.

#### 2.2.1. Frontier Molecular Orbital (FMO) Analysis

FMOs analysis is performed to get information about the electronic properties and reactivity of compounds. The energies of HOMOs (E_HOMOs_) and LUMOs (E_LUMOs_) explain electronic properties and HOMO–LUMO gaps (G_H-L_) validate kinetic stability and reactivity of compounds. Large G_H-L_ declared the less reactivity and more stability of compound and vice versa [39]. The energies of HOMOs (E_HOMOs_), energies of LUMOs, (E_LUMOs_), and HOMO–LUMO energy gaps (G_H-L_) of all compounds are summarized in Table 3.

Among the compounds **1a**–**1f**, **1d** has the smallest G_H-L_ (4.25 eV), whose E_HOMO_ and E_LUMO_ are −6.02 and −1.77 eV, respectively. Thus, **1d** is considered as kinetically less stable and moderately reactive among **1a**–**1f** series. Previously reported gaps for substituted benzimidazole-, thiazole-, and benzothiazole-based compounds range from 3 to 0.63 eV. They are reported as highly stable compounds [23,40,41]. On the other side, 4.69 eV of G_H-L_ is calculated for compounds **1a, 1b** and **1e**, which represent more stability and less reactivity of these compounds. The other compounds have G_H-L_ values of 4.25 to 4.52 eV, which represent their adequate stability with moderate reactivity. In the other series (**2a**–**2f**), **2d** has the lower G_H-L_ (4.79 eV), similar to the results of compound **1d**. The rest of the compounds have G_H-L_ values from 4.91 to 5.08 eV and represent high stability and less reactivity of these compounds. All compounds of this series have a larger G_H-L_ and are ultimately less reactive compared to compounds **1a**–**1f** (Table 3).

**Figure 4:**
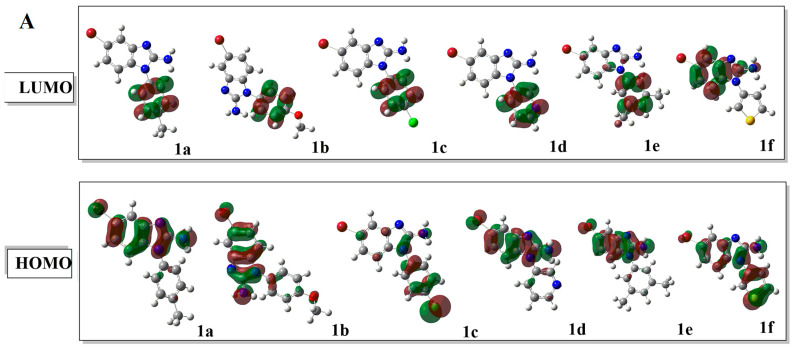

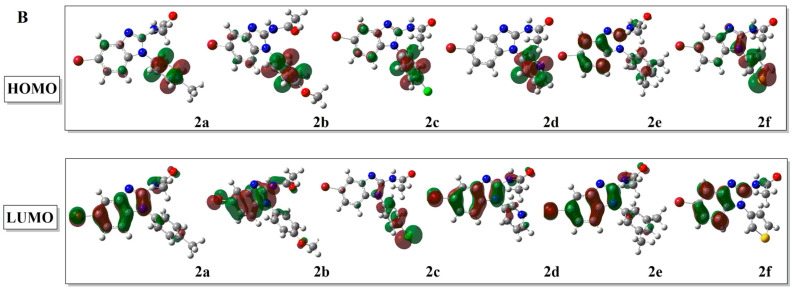
Isodensities of HOMOs and LUMOs of compounds (**A**) **1a**–**1f** and (**B**) **2a**–**2f** at cutoff values of 0.05.

The FMOs isodensity distributions of compounds **1a**–**1f** are shown in Figure 4A. The isodensity in HOMOs is distributed on benzimidazole rings and bromine atoms attached to these rings in compounds **1a** and **1e**. Bromine is a strong electron-withdrawing group so electronic densities move toward it. However, some of the isodensity also reside on oxygen, chlorine, nitrogen, and sulfur atoms of compounds **1b**–**d** and **1f**, respectively, because these atoms also have more electronegativity like bromine atoms. The result is the shift of electronic density from benzimidazole rings towards heteroatoms (C, O, N, S, and Cl) attached as substituents. This specific distribution explains the lower HOMO–LUMO gaps of these compounds (**1b**–**d** and **1f**) compared to other compounds (**1a** and **1e**). The isodensity shift is prominent for **1d** compared to other compounds. The isodensity in LUMOs is mainly distributed on phenyl rings in all compounds (**1a**–**1f,** except **1d**). These results clarify that the electron densities are migrating mainly from the donor (electron-donating groups terminal) to the acceptor unit (electron-withdrawing groups terminal). Compounds (**2a**–**2f**) also have similar isodensity distributions and their HOMO and LUMO densities are given in Figure 4B. Isodenisties are localized on the benzimidazole rings in compounds **2a**–**d** and **2e**. LUMO densities are present on the aromatic rings. In compounds **2c** and **2f**, HOMO densities shifted towards the chloro-substituted aromatic and thiophene rings and LUMO densities are on the benzimidazole rings. The gaps are mainly due to the smaller values of the LUMOs of **1d** and **2d**, and since the LUMOs are mainly centered on the N-arylated part, the gap is smaller due to the accepting nature of the pyridine. As shown by its highest electrophilicity value, the *ω* values of **1d** and **2d** are 3.56 and 3.80 eV, respectively.

#### 2.2.2. Reactivity Descriptor Parameters

For further interpretation of the reactivity of compounds (**1a**–**1f** and **2a**–**2f**), some other reactivity descriptor parameters were also analyzed and the results of their analysis are shown in Table 4. These parameters involve electron affinity (*E_A_*), ionization potential (*I*), electronic chemical potential (*μ*), chemical hardness (*η*), and electrophilicity index (*ω*). According to Koopman’s theorem, the negative values of HOMOs and LUMOs correspond to ionization potential (*I*) and electron affinity (*E_A_*), respectively [42,43,44].

The value of chemical hardness (*η*) is mathematically calculated as follows:

Chemical hardness (*η*) = (E_HOMO_ − E_LUMO_)/2

The chemical hardness of **1a**–**2f** compounds is from 2.13 to 2.35 eV. The highly unstable and most reactive compound is **1d** based on its small chemical hardness value (*η* = 2.13 eV). Compounds **1a, 1b,** and **1e** are stable compounds which are also confirmed from their *η* values of 2.34, 2.33, and 2.35 eV, respectively. The chemical hardness of other compounds is between 2.24 and 2.26 eV representing their moderate stability and reactivity. Ionization energy is between 5.78 and 6.02 eV and electron affinity are in the range of 1.10–1.77 eV. Looking towards compounds **2a**–**2f**, we see that **2d** has lower chemical hardness (2.39 eV), and others are noticed as hard compounds; their *η* values range from 2.45 to 2.54 eV. The ionization potential and electron affinity values of **2a**–**2f** are more than those of **1a**–**2f** (Table 4).

Electronic chemical potential (*μ*) tells about the charge transfer at their ground state inside the compounds, and it is mathematically represented as follows:

Electronic chemical potential (*μ*) = (E_HOMO_ + E_LUMO_)/2

where the *μ* of compounds **1a**–**1f** is from −3.44 to −3.89 eV. The highest *μ* value (−3.89 eV) and lowest *μ* value (−3.44 eV) were calculated for **1d** and **1e**, respectively. In other series (**1a**–**1f**), compound **2d** also has a high value of *μ* (−4.26 eV), which signifies that more charge transfer is from an electron donor to acceptor group.

Based on energy, the electrophilicity index (*ω*) represents the stability of compounds when an extra charge is transferred from the surrounding [44], and it is mathematically calculated as follows:

Electrophilicity index (*ω*) = *μ*^2^/2*η*

For compounds **1a**–**1f**, the *ω* values range from 3.61 to 4.02 eV. The compound **1d** has the highest *ω* value of 3.56 eV which indicates that it is less stable for the incoming charge. The presence of electron donor and acceptor groups linked through extended conjugation is the reason for less stability of this compound and enhance its reactivity as incoming charge causes more delocalization of electronic density. In the series of **2a**–**2f**, the *ω* value of **2d** is 3.80 eV and represents the more charge accepting capability of these compounds. Other compounds have a low *ω* value, i.e., up to 2.63 eV.

### 2.3. Non-Linear Optical (NLO) Properties

From the start of this century, scientists are following the methods to produce non-linear optical (NLO) materials because of their potential consumptions in optical data storage, optical communication, optical computing, optical limiting, medical imaging, laser devices, etc. [45,46]. To increase the NLO response of the compounds, different strategies are adopted, such as push–pull mechanism [47], metal–organic framework [48], excess electron system [45,49], etc. The organic molecules develop a strong NLO response as the transformation of electrons from donor to acceptor group is responsible for the improved value of *β*_o_ [50]. Polarizability (*α_o_*) and first hyperpolarizability (*β*_o_) parameters are used to measure the NLO response of respective compounds.

Polarizability (*α_o_*) and hyperpolarizability (*β*_o_) parameters of optical and nonlinear optical properties are studied at different density functionals, i.e., CAM-B3LYP, LC-BLYP, and ωB97XD (vide infra). CAM-B3LYP has a 0.65 fraction of nonlocal exchange at an asymptotic distance and is a highly reliable method for calculating hyperpolarizabilities [36]. Full range-separated functionals LC-BLYP [37] and ωB97XD [38] have the correct 1.00 fraction of nonlocal exchange they are also well-known funtionals in the field of NLO. Almost comparable results are obtained at all selected density functionals where polarizability ranges from 182 to 239 au and hyperpolarizability is between 1.73 × 10^−30^ and 5.63 × 10^−30^ esu for **1a**–**1f** and **2a**–**2f** at LC-BLYP. Polarizability values for all them (**1a**–**1f** and **2a**–**2f**) is from 188 to 246 au and hyperpolarizability is between 5.31 × 10^−30^ and 1.90 × 10^−30^ esu at ωB97XD. Moreover, 188 to 247 au and 2.00 × 10^−30^ to 5.66 × 10^−30^ esu are the polarizability and the hyperpolarizability ranges for **1a**–**1f** and **2a**–**2f** at CAM-B3LYP (Table 3 and Supplementary Materials Table S1). The highest polarizability (247 au) and hyperpolarizability (5.66 × 10^−30^ esu) are calculated at CAM-B3LYP functional. Therefore, the results of CAM-B3LYP are given in the main manuscript other results are given in supplementary material (Table S1). The short-range intermolecular interaction plays important role in describing optical and nonlinear optical properties of all these compounds due to which the results of CAM-B3LYP are better than the other two functionals.

Polarizability is the distribution of electron density in a system. The compounds having electron-donating and accepting groups at the opposite edge of phenyl rings have a high value of *α*_o_ because of positive and negative centers. On the other side, the compounds having equal electronic density distribution due to similar functional groups on the opposite terminal have a low *α*_o_ value. The compound **1a** has a high value of *α*_o_ (220 au) whereas **1e** has a low *α*_o_ value (182 au). Other compounds have moderate *α*_o_ values, from 183 to 208 au. The *α*_o_ values of compounds **2a**–**2f** range from 208 to 239 au. The *α*_o_ value of **2e** is high due to electron-withdrawing and donating groups attached at opposite terminals of the ring.

Benzimidazole ring act as electron-rich species when phenyl ring having different substitution are attached. The shifting of electronic density occurs due to an extended conjugated system between electron-withdrawing and donating groups, the corresponding intersystem charge transfer (ICT) increases the *β*_o_. The compound **1b** displayed the largest *β*_o_ value (5.66 × 10^−30^ esu), because of the strong electron-donating group (MeO-) at the para position of the aromatic ring, and on the other side of this molecule, the electron-withdrawing bromine group is attached. According to the push–pull mechanism, shifting of electronic density (ICT) occur under extended conjugation. A *β*_o_ value of (4.61 × 10^−30^ esu) was observed for compound **1a** where a lower electron-donating methyl group is attached to the phenyl ring compared to the methoxy group. The *β*_o_ values of **1d**, **1e**, and **1f** compounds are 4.40 × 10^−30^, 4.00 × 10^−30^, and 4.30 × 10^−30^ esu, respectively. All these compounds have electron-donating property-based substations, i.e., pyridine, di-methylbenzene, and thiazole rings, which enhance *β*_o._ In compound **1c**, chlorine and bromine groups are present on opposite terminals of the compound and result in a lower *β*_o_ value (3.81 × 10^−30^ esu). Both halogen groups withdraw electronic density towards themselves, and the ICT transfer is low compared to other above-discussed compounds. For compounds **2a**–**2f**, *β*_o_ values are in a range from 2.00 × 10^−30^ to 3.54 × 10^−30^ esu. Compounds (**2a**–**2f**) have a lower *β*_o_ compared to compounds (**1a**–**2f)**, but the overall results are similar **1a**–**2f** series. The reason is the acetamide group where resonance occurs between the nitrogen and carbonyl groups, and it also acts as an electron-donating group, although the extent of donation is low. Strong electron-donating methoxy of **2b** (3.54 × 10^−30^ esu), moderate electron-donating pyridine, and thiophene groups of **2d** (2.40 × 10^−30^ esu**)** and **2f** (2.37 × 10^−30^ esu) enhance delocalization of the π bonding and electron-withdrawing group bromine on the other side accept the electronic density. The shifting of density is more in the case of methoxy group as compared to thiophene and pyridine. The sulfur of thiophene (**2f**) has the opposite effect due to the electronegativity of sulfur, so its *β*_o_ is lower than the methoxy group. A low electron-donating group methyl in compounds **2a** (2.02 × 10^−30^ esu), **2e** (2.01 × 10^−30^ esu) has a lower tendency to undergo ICT and have comparatively low *β*_o_. The lowest *β*_o_ value is obtained for **2c** due to electron-withdrawing groups’ attachment on the opposite side but due to the presence of moderately electron-donating group the ICT is more compared to **1c** and a value of 2.00 × 10^−30^ is seen.

We compare the result of **1b** at CAM-B3LYP with already reported compounds from benzimidazole family and their comparative graph is given in Table 5. The table clearly represents that values of our designed compounds are close to the already reported compounds (salicylidenephenyl)benzimidazole] (**Spbzl**) at B3LYP, salicylidenephenyl)benzimidazole]* (Spbzl*) at CAM-B3LYP and 4-((3-(1H-benzimidazol-2-yl)phenylimino)methyl)-3-hydroxybenzoic acid (**Pbzlb**) at CAM-B3LYP.) in the gaseous phase at B3LYP and CAM-B3LYP level [51]. The table shows almost similar NLO response of our compounds to already reported on which justifies the better performance of our compounds.

### 2.4. UV–VIS Absorption Analysis

**Figure 5:**
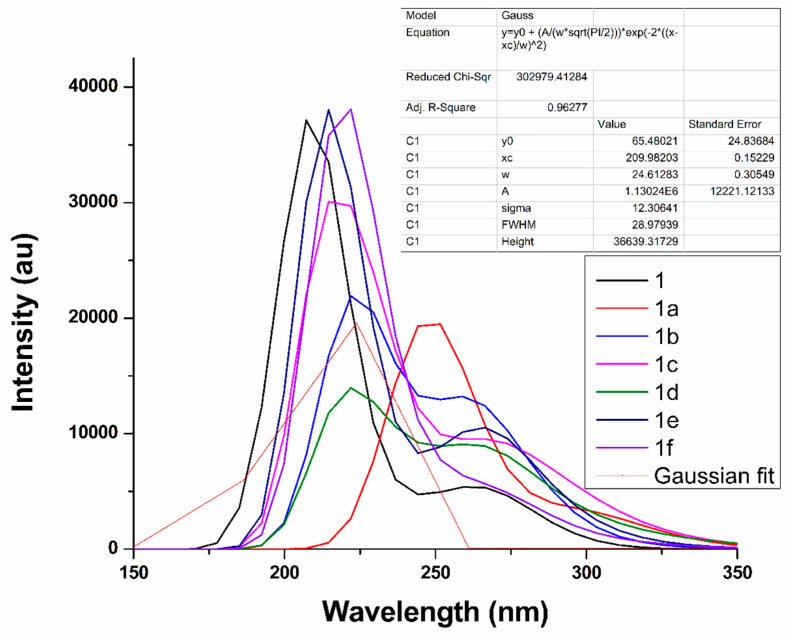

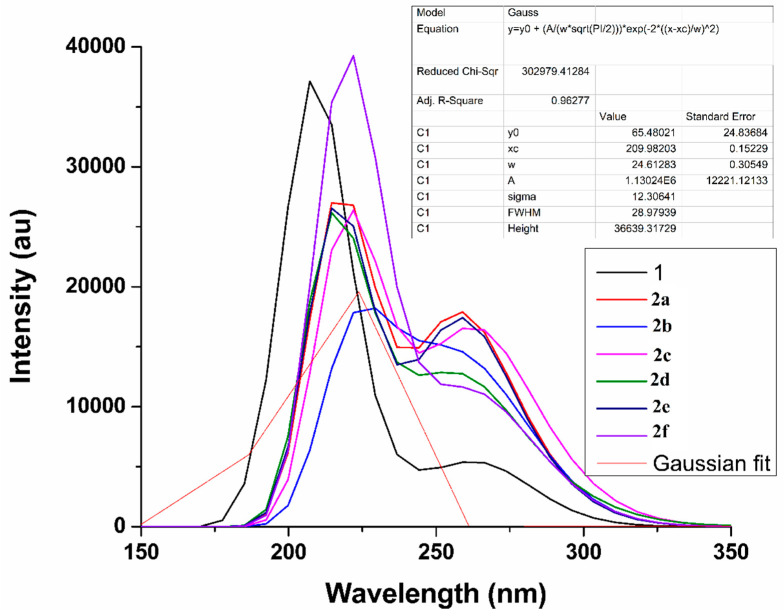
Calculated UV–VIS absorption spectra of parent (**1**) and substituted compounds (**1a**–**1f** (above) and **2a**–**2f** (below)), using GaussView 05 software. The width at half maximum used for the Gaussian broadening is 28.98 *σ*.

UV–VIS absorption analysis is performed by using the TD-DFT method to get an insight into electronic excitation that occurs from a lower energy state to a higher energy state [52]. UV–VIS spectra of the parent compound (**1**) and substituted compounds (**1a**–**2f** and **2a**–**2f**) are given in Figure 5. Results of oscillating strength (*f*_o_), excitation energies (∆E), and wavelength (λ) of all compounds (**1**,**1a**–**2f** and **2a**–**2f**) are given in Table 6 The redshift is seen in all substituted compounds (**1a**–**2f**) in comparison to the parent compound (**1**). The pronounced effect is seen for **1a** compound in series of **1a**–**2f** compounds. The electronic excitation takes place from donor–acceptor groups. In compounds where on both sides of the parent skeleton electron-donating and withdrawing groups are attached, this excitation is more prominent. In compound **1a**, the electron-withdrawing bromine group (-Br) is attached, while, on the other side, the electron-donating methyl group (-CH_3_) is attached. A prominent redshift of 248 nm wavelength is observed for it (**1a**). Based on the electron-donating capability of other groups, the corresponding redshift is observed. The increasing trend of wavelength is as follows: **1a** (248 nm) > **1b** (226 nm) > **1d** (221 nm) > **1f** (217 nm) > **1e** (213 nm) > **1c** (212 nm).

UV–VIS analysis for **2a**–**2f** series of compounds is also performed where hydrogen is replaced by an amide group (Figure 5). Again, a redshift is seen compared to parent compound **1**, which indicates the electronic excitation; the bathochromic shift is seen where peaks move toward a higher wavelength. Maximum wavelength is obtained for compound **2b**, where the peak is located at 222 nm. The amide group increases the wavelength of the compounds that have a lower wavelength in the **2a**–**2f** series, because of the presence of electron-withdrawing groups at both opposite terminals (compound **2e**). The maximum wavelength for these compounds **2a**–**2f** ranges from 214 to 223 nm. Amide is an electron-withdrawing group, and it facilitates the push–pull mechanism and delocalization of electrons. The observed electronic excitations enable these compounds (**1a**–**1f** and **2a**–**2f**) as efficient NLO materials usage in a second-harmonic generation. These compounds show transparency below 200 nm, so they can also be used in UV laser technology. Su and co-workers also worked on the single group substitution of benzimidazole; they observed that electron-donating groups decrease the wavelength, whereas the electron-withdrawing group increases the observed wavelength [53]. Their calculated wavelength is lower than our reported maximum wavelength for compounds **1a** and **1b**.

## 3. Experimental

All the purchased chemicals (Sigma Aldrich, St. Louis, MO, USA) and solvents were purified before use by distillation, or, for extra purity, some of the solvents were also dried in the lab. All the reactions were completed under and open-air environment. The reaction progress was checked by using a TLC card. Organic solutions were evaporated from the reaction by using a rotatory evaporator (Buchi, R-210, Allschwil, Switzerland) and vacuum pump (Buchi, V-700, Flawil, Switzerland). NMR spectra were calculated in CDCl_3_ and DMSO-*d*_6_ by using Bruker ARX 400 and 125 MHz FT–NMR spectrometers (Billercia, MA, USA). Different aryl boronic acid, bases, and solvents were used for the synthesis of required compounds by following specific methods.

### 3.1. General Protocol for the Synthesis of Compounds

#### 3.1.1. General Procedures for the Synthesis of 5-Bromo-1*H*-benzo[*d*]imidazol-2-amine Derivatives (**1a**–**1f**)

A dry 100 mL round-bottom flask was loaded with 0.94 mmol of 5-bromo-2-aminobenzimidazole (**1**), 0.94 mmol of Cu(OAc)_2_, and 8 mL of methanol. Then the mixture was stirred under an open-air environment for 15 min. After that, 1.88 mmol of base Et3N or TMEDA, 1.128 mmol of aryl boronic acid, and 2 mL of water were added and stirred again for two hours. The reaction progress was monitored by TLC. After completion, the reaction mixture was filtered and concentrated by rotary evaporated to get residue [29]. The residue was column chromatographed to get the desired product, which was further analyzed by using NMR spectroscopic techniques.

#### 3.1.2. Synthesis of *N*-(5-Bromo-1*H*-benzo[*d*]imidazol-2-yl) Acetamide (**2**)

The mixture of 5-bromo-2-aminobenzimidazole (0.1 g, 0.47 mmol) in 5 mL of acetic anhydride was heated at 40 °C for 4 h. The precipitates of the reaction mixture were cooled to 0 °C, then filtered off and washed with DCM and water. Finally, they were dried to give the final product with white crystals [28].

#### 3.1.3. General Procedures for the Synthesis of *N*-(5-Bromo-1*H*-benzo[*d*]imidazol-2-yl) Acetamide Derivatives (**2a**–**2f**)

A dry 100 mL round-bottom flask was loaded with 0.39 mmol of *N*-(5-bromo-1*H*-benzo[*d*]imidazol-2-yl)acetamide, 0.47 mmol of aryl boronic acid, 0.59 mmol of Cu(OAc)_2_, 0.59 mmol of triethylamine, 100 mg of 4 Å molecular sieves, and then 10 mL of dry DCM. Then, the reaction mixture was stirred at room temperature, for 36 h; TLC examination was carried out to monitor the reaction completion. Upon completion, the reaction mixture was filtered and washed with ethyl acetate (5 mL). Then, the solvent was evaporated by rotary evaporation to obtain the residue [15,30]. The residue was column chromatographed by utilizing a mixture of hexane and ethyl acetate (60/40) to obtain the wanted product with a good % yield, which was further analyzed by using NMR spectroscopic techniques. The NMR spectra are given in Supplementary Material (Figures S1–S18).

### 3.2. Characterization Data

*5-bromo-1-p-tolyl-1H-benzo[d]imidazol-2-amine* (**1a**): ^1^H-NMR (400 MHz, CDCl_3_): δ = 7.86 (d, *J* = 6.9 Hz, 3H), 7.18 (d, *J* = 7.5 Hz, 3H), 7.12 (d, *J* = 8.0 Hz, 1H), 6.98 (s, 2H), 2.34 (s, 3H). ^13^C-NMR (125 MHz, DMSO-*d*_6_): δ = 158.42, 142.54, 140.36, 138.02, 137.53, 128.95, 128.85, 128.22, 127.79, 124.96, 119.63, 112.46, 111.18, 21.15. Anal. Calcd. For C_14_H_12_BrN_3_: C, 55.65; H, 4.00; N, 13.91. Found: C, 55.72; H, 4.09; N, 13.88.

*5-bromo-1-(4-methoxyphenyl)-1H-benzo[d]imidazol-2-amine* (**1b**): ^1^H-NMR (400 MHz, CDCl_3_): δ = 7.93 (d, *J* = 7.9 Hz, 2H), 7.70 (d, *J* = 29.8 Hz, 1H), 7.08 (d, *J* = 7.4 Hz, 1H), 6.94–6.91 (m, 3H), 6.82 (s, 2H), 3.80 (s, 3H). ^13^C-NMR (125 MHz, DMSO-*d*_6_): δ = 155.58, 140.21, 140.14, 134.50, 130.67, 124.58, 121.64, 1117.64, 116.91, 113.68, 110.82, 110.77, 109.87, 60.33. Anal. Calcd. For C_14_H_12_BrN_3_O: C, 52.85; H, 3.80; N, 13.21. Found: C, 52.83; H, 3.86; N, 13.25.

*5-bromo-1-(3-chlorophenyl)-1H-benzo[d]imidazol-2-amine* (**1c**): ^1^H-NMR (400 MHz, CDCl3) δ = 7.85 (d, *J* = 7.9 Hz, 3H), 7.63 (s, 1H), 7.33 (d, *J* = 8.2 Hz, 4H), 7.22 (d, *J* = 6.8 Hz, 1H). ^13^C-NMR (125 MHz, DMSO-d6): δ = 160.87, 156.29, 150.14, 141.45, 139.32, 139.20, 137.30, 134.46, 121.11, 118.66, 114.38, 112.41, 110.88. Anal. Calcd. For C13H9BrClN3: C, 48.40; H, 2.81; N, 13.03. Found: C, 48.48; H, 2.85; N, 13.00.

*5-bromo-1-(pyridin-3-yl)-1H-benzo[d]imidazol-2-amine* (**1d**): ^1^H-NMR (400 MHz, CDCl_3_) δ = 8.60 (d, *J* = 4.2 Hz, 2H), 7.66 (t, *J* = 7.7 Hz, 2H), 7.41 (s, 1H), 7.14 (dt, *J* = 16.8, 8.4 Hz, 4H). ^13^C-NMR (125 MHz, DMSO-*d*_6_): δ = 161.80, 146.93, 136.70, 135.23, 134.89, 134.53, 133.57, 131.78, 121.71, 119.30, 117.42, 114.69. Anal. Calcd. For C_12_H_9_BrN_4_: C, 49.85; H, 3.14; N, 19.38. Found: C, 49.93; H, 3.15; N, 19.33.

*5-bromo-1-(3,5-dimethylphenyl)-1H-benzo[d]imidazol-2-amine* (**1e**): ^1^H-NMR (400 MHz, CDCl_3_) δ = 7.68–7.47 (m, 5H), 7.32 (s, 1H), 7.07 (s, 2H), 2.37 (s, 6H). ^13^C-NMR (125 MHz, DMSO-*d*_6_): δ = 154.26, 151.85, 149.37, 142.11, 136.30, 130.94, 126.54, 120.85, 118.15, 116.85, 113.22, 111.00, 27.46. Anal. Calcd. For C_15_H_14_BrN_3_: C, 56.98; H, 4.46; N, 13.29. Found: C, 57.02; H, 4.49; N, 13.24.

*5-bromo-1-(thiophen-3-yl)-1H-benzo[d]imidazol-2-amine* (**1f**): ^1^H-NMR (400 MHz, CDCl_3_): δ = 7.82 (s, 1H), 7.49 (t, *J* = 9.7 Hz, 2H), 7.37–7.32 (m, 2H), 7.12 (d, *J* = 7.3 Hz, 1H), 6.98 (s, 2H). ^13^C-NMR (125 MHz, DMSO-*d*_6_): δ = 154.67, 149.45, 144.50, 129.31, 122.00, 120.36, 117.72, 116.02, 115.76, 115.01, 111.88. Anal. Calcd. For C_11_H_8_BrN_3_S: C, 44.91; H, 2.74; N, 14.28. Found: C, 44.98; H, 2.76; N, 14.25.

*N-(5-bromo-1H-benzo[d]imidazol-2-yl)acetamide* (**2**): ^1^H-NMR (400 MHz, CDCl_3_): δ = 7.98 (d, *J* = 2.0 Hz, 1H), 7.62 (dd, *J* = 9.2, 2.8 Hz, 2H), 6.97 (s, 1H), 5.94 (s, 1H), 2.28 (s, 3H). ^13^C-NMR (125 MHz, CDCl_3_): δ = 172.48, 147.28, 134.47, 133.26, 122.63, 117.39, 115.46, 112.57, 23.43. Anal. Calcd. For C_9_H_8_BrN_3_O: C, 42.54; H, 3.17; N, 16.54. Found: C, 42.59; H, 3.20; N, 16.52.

*N-(5-bromo-1-p-tolyl-1H-benzo[d]imidazol-2-yl)acetamide* (**2a**): ^1^H-NMR (400 MHz, CDCl_3_): δ = 8.08 (d, *J* = 8.1 Hz, 1H), 7.85 (d, *J* = 8.1 Hz, 1H), 7.68 (d, *J* = 8.1 Hz, 2H), 7.31 (d, *J* = 8.1 Hz, 1H), 7.22 (d, *J* = 8.2 Hz, 2H), 4.37 (s, 1H), 2.52 (s, 3H), 2.47 (s, 3H). ^13^C-NMR (125 MHz, CDCl_3_): δ = 172.46, 148.04, 139.77, 137.59, 135.30, 133.73, 130.69, 125.23, 120.74, 118.42, 115.78, 113.78, 23.91, 20.92. Anal. Calcd. For C_16_H_14_BrN_3_O: C, 55.83; H, 4.10; N, 12.21. Found: C, 55.91; H, 4.14; N, 12.19.

*N-(5-bromo-1-(4-methoxyphenyl)-1H-benzo[d]imidazol-2-yl)acetamide* (**2b**): ^1^H-NMR (400 MHz, CDCl_3_): δ = 7.97 (d, *J* = 1.6 Hz, 1H), 7.84 (d, *J* = 8.7 Hz, 2H), 7.66 (d, *J* = 8.8 Hz, 1H), 7.41 (dd, *J* = 8.4, 1.2 Hz, 1H), 7.0 (d, *J* = 8.0 Hz, 2H), 5.91 (s, 1H), 3.88 (s, 3H), 2.24 (s, 3H). ^13^C-NMR (125 MHz, CDCl_3_): δ = 171.98, 162.78, 148.75, 138.98, 135.39, 130.71, 128.74, 123.64, 120.62, 118.89, 116.32, 113.01, 57.46, 23.86. Anal. Calcd. For C_16_H_14_BrN_3_O_2_: C, 53.35; H, 3.92; N, 11.67. Found: C, 53.42; H, 3.90; N, 11.66.

*N-(5-bromo-1-(3-chlorophenyl)-1H-benzo[d]imidazol-2-yl)acetamide* (**2c**): ^1^H-NMR (400 MHz, CDCl_3_): δ = 8.04–8.02 (m, 1H), 7.94 (d, *J* = 1.8 Hz, 1H), 7.81 (d, *J* = 1.7 Hz, 1H), 7.64 (d, *J* = 8.5 Hz, 1H), 7.47–7.41 (m, 3H), 5.97 (s, 1H), 2.28 (s, 3H). ^13^C-NMR (125 MHz, CDCl_3_): δ = 172.67, 148.47, 138.78, 135.56, 134.58, 132.67, 130.77, 128.57, 126.82, 124.04, 122.09, 120.02, 117.47, 113.89, 24.01. Anal. Calcd. For C_15_H_11_BrClN_3_O: C, 49.41; H, 3.04; N, 11.52. Found: C, 49.43; H, 3.09; N, 11.49.

*N-(5-bromo-1-(pyridin-3-yl)-1H-benzo[d]imidazol-2-yl)acetamide* (**2d**): ^1^H-NMR (400 MHz, DMSO-*d*_6_): δ = 8.17 (d, *J* = 2.3 Hz, 1H), 7.96 (d, *J* = 7.4 Hz, 2H), 7.80 (dd, *J* = 8.8, 2.4 Hz, 2H), 7.55 (d, *J* = 8.8 Hz, 2H), 4.70 (s, 1H), 2.55 (s, 3H). ^13^C-NMR (125 MHz, CDCl_3_): δ = 171.94, 147.92, 145.46, 142.56, 136.84, 134.87, 131.75, 129.99, 125.45, 122.04, 118.52, 117.15, 115.97, 23.67. Anal. Calcd. For C_14_H_11_BrN_4_O: C, 50.77; H, 3.35; N, 16.92. Found: C, 50.86; H, 3.41; N, 16.86.

*N-(5-bromo-1-(3,5-dimethylphenyl)-1H-benzo[d]imidazol-2-yl)acetamide* (**2e**): ^1^H-NMR (400 MHz, CDCl_3_) δ = 7.67 (s, 3H), 7.34 (s, 1H), 7.11 (d, *J* = 6.4 Hz, 3H), 2.34 (s, 3H), 2.32 (s, 6H). ^13^C-NMR (125 MHz, DMSO-*d*_6_): δ = 172.65, 149.19, 136.09, 134.71, 132.25, 129.28, 127.51, 126.28, 124.61, 121.69, 117.05, 113.96, 21.51, 17.65. Anal. Calcd. For C_17_H_16_BrN_3_O: C, 57.00; H, 4.50; N, 11.73. Found: C, 57.06; H, 4.53; N, 11.70.

*N-(5-bromo-1-(thiophen-3-yl)-1H-benzo[d]imidazol-2-yl)acetamide* (**2f**): ^1^H-NMR (400 MHz, CDCl_3_) δ = 7.98 (d, *J* = 7.2 Hz, 2H), 7.84 (s, 1H), 7.63–7.59 (m, 1H), 7.43 (s, 1H), 7.36 (s, 1H), 4.37 (s, 1H), 2.34 (s, 3H). ^13^C-NMR (125 MHz, DMSO-*d*_6_): δ = 172.75, 149.97, 148.28, 135.52, 131.88, 125.49, 122.28, 121.86, 114.90, 113.05, 111.91, 109.59, 21.51. Anal. Calcd. For C_13_H_10_BrN_3_OS: C, 46.44; H, 3.00; N, 12.50. Found: C, 46.49; H, 2.98; N, 12.56.

### 3.3. Computational Details

All synthesized compounds (**1a**–**1f** and **2a**–**2f**) were computationally studied by using Gaussian 09 [33] and GaussView 5.0 software [54]. First of all, compounds were optimized at B3LYP functional of DFT. B3LYP functional is widely used for optimization because of fewer chances of errors in the geometrical parameters’ description [35,55]. Frequency analyses were done at the same level of theory for further confirmations of these structures as true minima energy structures on potential energy surfaces. An analysis of frontier molecular orbitals (FMOs) was performed, and reactivity descriptor parameters were calculated at B3LYP/6-31+G(d,p) method. Nonlinear optical (NLO) properties, including polarizabilities and first hyperpolarizability parameters, were measured at CAM-B3LYP [36], LC-BLYP [37], and ωB97XD [38] with a 6-31+G(d,p) [34] level.

## 4. Conclusions

Consequently, our investigation concentrated on the cross-coupling of 5-bromo-2-aminobenzimidazole and aryl boronic acids under various reaction conditions. The impact of different bases and solvents was observed, and we noted that the reaction is not potent with Et_3_N and pyridine; however, the reaction products obtained a good yield with TMEDA. Finally, all synthesized derivatives (**1a**–**1f** and **2a**–**2f**) were computationally studied by applying DFT calculations. Frontier molecular orbital (FMO) analysis provides information about the electronic properties and reactivity of compounds. Out of **1a**–**1f**, compound **1d,** and out of **2a**–**2f**, compound **2d** presented the lowest HOMO–LUMO energy gap of 4.25 and 4.79 eV, respectively. The isodensity shift was more pronounced for these compounds as compared to others. Reactivity descriptor parameters were also calculated to determine the chemical reactivity relations of all synthesized derivatives. Compounds **1d** and **2d** have the lowest values of chemical hardness (2.13 and 2.39 eV), lower electronic chemical potential (−3.89 and −4.26 eV), and highest values of electrophilicity index (3.56 and 3.80 eV), respectively. Also, Compounds **2d** has lowest electronic chemical potential (−4.26 eV) among all compounds. The compounds **1a**–**1f** displayed better results for all properties when compared to compounds **2a**–**2f**, because the acetamide group shows conjugation between nitrogen and carbonyl groups, where electronic density shifting is low. Moreover, polarizability (α_o_) and first hyperpolarizability (β_o_) parameters were used to measured NLO response. Overall, **1a** has a high αo value of 228 au, and **2e** exhibited the highest value of 247 au due to electron-withdrawing and donating groups attached at opposite terminals of the rings. The highest value of βo is shown by 1b (5.66 × 10^−30^ au). UV–VIS absorption analysis is performed to understand electronic excitation in the designed compounds. These results suggested the use of these compounds in the field of optics and nonlinear optics.

## Figures and Tables

**Table 1:** Optimization of Cu(II)-catalyzed C-N cross-coupling of 5-bromo-2-amino benzimidazole and aryl boronic acid in MeOH/H_2_O at room temperature.

Entry	Aryl Boronic Acid	Base	Product	Yield (%)
**1**	4-MeC_6_H_4_B(OH)_2_	Et_3_N	**1a**	trace
**2**	4-MeC_6_H_4_B(OH)_2_	TMEDA	**1a**	70
**3**	4-OMeC_6_H_4_B(OH)_2_	TMEDA	**1b**	80
**4**	3-ClC_6_H_4_B(OH)_2_	TMEDA	**1c**	78
**5**	3-NC_5_H_4_B(OH)_2_	TMEDA	**1d**	73
**6**	3,5-Me_2_C_6_H_3_B(OH)_2_	TMEDA	**1e**	71
**7**	3-SC_4_H_3_B(OH)_2_	TMEDA	**1f**	69

**Table 2:** Optimization of Cu(II)-catalyzed C-N cross-coupling of 5-bromo-1*H*-benzo[*d*]imidazol-2-amine and aryl boronic acid in DCM at room temperature.

Entry	Aryl Boronic Acid	Base	Product	Yield (%)
**1**	4-MeC_6_H_4_B(OH)_2_	Et_3_N	**2a**	40
**2**	4-MeC_6_H_4_B(OH)_2_	Pyridine	**2a**	37
**3**	4-OMeC_6_H_4_B(OH)_2_	Et_3_N	**2b**	42
**4**	3-ClC_6_H_4_B(OH)_2_	Et_3_N	**2c**	41
**5**	3-NC_5_H_4_B(OH)_2_	Et_3_N	**2d**	45
**6**	3,5-Me_2_C_6_H_3_B(OH)_2_	Et_3_N	**2e**	49
**7**	3-SC_4_H_3_B(OH)_2_	Et_3_N	**2f**	39

**Table 3:** Energy of HOMOs (E_HOMOs_) and LUMOs (E_LUMOs_), HOMO-LUMO energy gap (G_H-L_), dipole moment (*μ*_o_), polarizability (*α_o_*), and first static hyperpolarizability (*β*_o_) of compounds **1a**–**1f** and **2a**–**2f**.

Compounds	E_HOMO_ (eV)	E_LUMO_ (eV)	G_H-L_ (eV)	*μ*_o (_Debye)	*α*_o_ (Au)	*β*_o_ (Esu)
Density Functionals	B3LYP				CAM-B3LYP	
**1a**	−5.81	−1.12	4.69	8.46	228	4.61 × 10^−30^
**1b**	−5.78	−1.11	4.66	7.95	215	5.66 × 10^−30^
**1c**	−5.99	−1.50	4.49	4.91	210	3.81 × 10^−30^
**1d**	−6.02	−1.77	4.25	5.63	190	4.40 × 10^−30^
**1e**	−5.79	−1.10	4.69	7.56	222	4.00 × 10^−30^
**1f**	−5.91	−1.39	4.52	6.32	188	4.30 × 10^−30^
**2a**	−6.44	−1.40	5.04	6.05	235	2.02 × 10^−30^
**2b**	−6.19	−1.11	5.08	5.93	240	3.54 × 10^−30^
**2c**	−6.59	−1.68	4.91	3.74	236	2.00 × 10^−30^
**2d**	−6.66	−1.87	4.79	5.35	215	2.40 × 10^−30^
**2e**	−6.42	−1.39	5.03	6.03	247	2.01 × 10^−30^
**2f**	−6.52	−1.52	5.00	4.69	215	2.37 × 10^−30^

**Table 4:** Ionization potential (*I*), electron affinity (*E_A_*), chemical hardness (*η*), electronic chemical potential (*μ*), and electrophilicity index (*ω*) of compounds **1a**–**1f** and **2a**–**2f**.

Compounds	*I* (eV)	*E_A_* (eV)	*η* (eV)	*μ* (eV)	*ω* (eV)
**1a**	5.81	1.12	2.34	−3.47	2.56
**1b**	5.78	1.11	2.33	−3.45	2.55
**1c**	5.99	1.50	2.24	−3.75	3.12
**1d**	6.02	1.77	2.13	−3.89	3.56
**1e**	5.79	1.10	2.35	−3.44	2.53
**1f**	5.91	1.39	2.26	−3.65	2.94
**2a**	6.44	1.40	2.52	−3.92	3.04
**2b**	6.19	1.11	2.54	−3.65	2.63
**2c**	6.59	1.68	2.45	−4.14	3.49
**2d**	6.66	1.87	2.39	−4.26	3.80
**2e**	6.42	1.39	2.52	−3.90	3.03
**2f**	6.52	1.52	2.50	−4.02	3.23

**Table 5:** Hyperpolarizabilty values of currently reported compound **1b** at CAM-B3LYP, and already reported salicylidenephenyl)benzimidazole] (**Spbzl**) at B3LYP, salicylidenephenyl)benzimidazole]* (Spbzl*) at CAM-B3LYP and 4-((3-(1H-benzimidazol-2-yl)phenylimino)methyl)-3-hydroxybenzoic acid (**Pbzlb**) at CAM-B3LYP.

Compounds	Hyperpolarizability (*β*_o_)
**1b**	5.66 × 10^−30^
**Spbzl**	1.27 × 10^−29^
**Spbzl**	8.50 × 10^−30^
**Pbzlb**	1.48 × 10^−29^

**Table 6:** Oscillating strength (*f*_o_), excitation energies (∆E) and wavelength (*λ***_max_**) of parent and substituted compounds (**1**,**1a**–**2f** and **2a**–**2f**).

Compounds	*f*_o_	∆E	*λ*_max_
**1**	0.71	5.94	209
**1a**	0.16	5.00	248
**1b**	0.32	5.49	226
**1c**	0.48	5.85	212
**1d**	0.18	5.62	221
**1e**	0.65	5.82	213
**1f**	0.45	5.71	217
**2a**	0.40	5.69	218
**2b**	0.14	5.56	223
**2c**	0.27	5.70	218
**2d**	0.48	5.78	214
**2e**	0.37	5.74	216
**2f**	0.31	5.67	219

## Data Availability

Not applicable.

## Supplementary Materials

The following are available online. Figures S1–S18: ^1^H-NMR and s ^13^C-NMR pectrum of compound **1a**–**2f**; Table S1: Polarizability and hyperpolarizability of compounds **1a**–**1f** and **2a**–**2f**.

## Sample Availability

Samples of the compounds are available from the authors.
