# Supplementary material for: N-Arylation of Protected and Unprotected 5-Bromo-2-aminobenzimidazole as Organic Material: Non-Linear Optical (NLO) Properties and Structural Feature Determination through Computational Approach
Source: Molecules. 2021 Nov 17;26(22):6920. doi: 10.3390/molecules26226920 (PMC8624707; doi:10.3390/molecules26226920)
Supplement: Supplementary file 1 [file molecules-26-06920-s001.zip › molecules-1304666-supplementary.pdf]

Supplementary Data

N-Arylation of protected & unprotected 5-bromo 2-amino benzoimidazole as Organic material: Non-linear optical (NLO) properties and structural feature determination through computational approach

Mubeen Mumtaz [1], Nasir Rasool [1,*], Gulraiz Ahmad [1], Naveen Kosar [2,3], Umer Rashid [4,*]

[1] Department of Chemistry, Government College University, Faisalabad 38000, Pakistan

[2] Department of Chemistry, University of Management and Technology (UMT), C11, Johar Town Lahore, Pakistan

[3] Department of Chemistry, COMSATS University Islamabad, Abbottabad Campus, Abbottabad, Pakistan

[4] Institute of Advanced Technology, University Putra Malaysia, 43400 UPM Serdang, Selangor, Malaysia

* Corresponding authors E-mail addresses:

Nasir Rasool Email: nasirrasool@gcuf.edu.pk

Umer Rashid Email:umer.rashid@upm.edu.my

Table of Contents:

Figure S1: ^{1}H NMR spectrum of compound **1a** S3

Figure S2: ^{13}C NMR spectrum of compound **1a** S3

Figure S3: ^{1}H NMR spectrum of compound **1b** S4

Figure S4: ^{13}C NMR spectrum of compound **1b** S4

Figure S5: ^{1}H NMR spectrum of compound **1c** S5

Figure S6: ^{13}C NMR spectrum of compound **1c** S5

Figure S7: ^{1}H NMR spectrum of compound **1d** S6

Figure S8: ^{13}C NMR spectrum of compound **1d** S6

Figure S9: ^{1}H NMR spectrum of compound **1e** S7

Figure S10: ^{13}C NMR spectrum of compound **1e** S7

Figure S11: ^{1}H NMR spectrum of compound **1f** S8

Figure S12: ^{13}C NMR spectrum of compound **1f** S8

Figure S13: ^{1}H NMR spectrum of compound **2a** S9

Figure S14: ^{1}H NMR spectrum of compound **2d** S9

Figure S15: ^{1}H NMR spectrum of compound **2e** S10

Figure S16: ^{13}C NMR spectrum of compound **2e** S10

Figure S17: ^{1}H NMR spectrum of compound **2f** S11

Figure S18: ^{13}C NMR spectrum of compound **2f** S11

Table S1: Polarizability and hyperpolarizability of compounds **1a-1f** and **2a-2f** **S12**

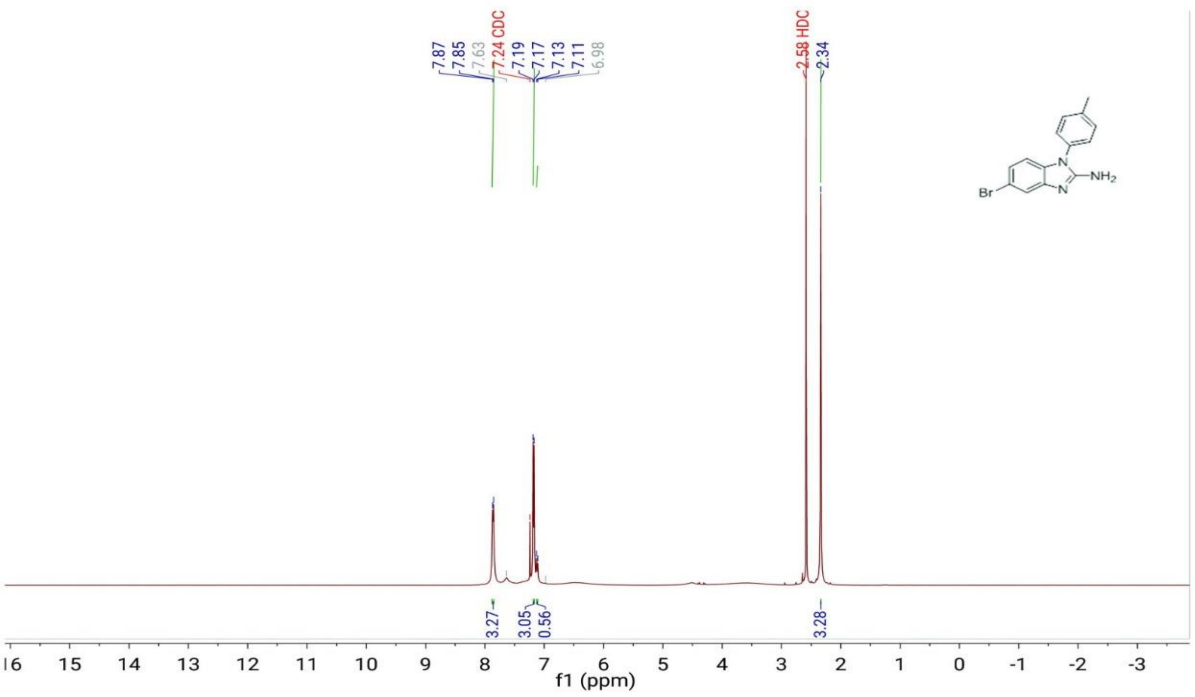

Figure S1. ^{1}H NMR (400 MHz, $CDCl_3$) of compound **1a**.

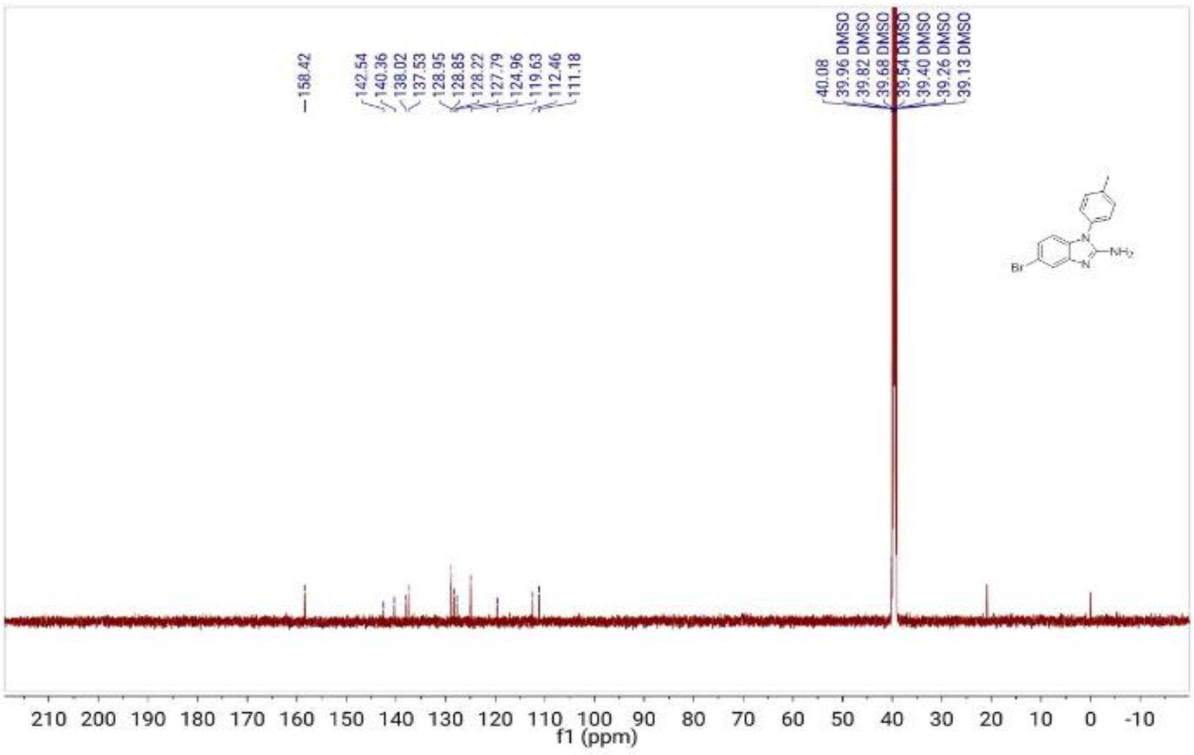

Figure S2. ^{13}C NMR (125 MHz, DMSO-d_6) of compound **1a**.

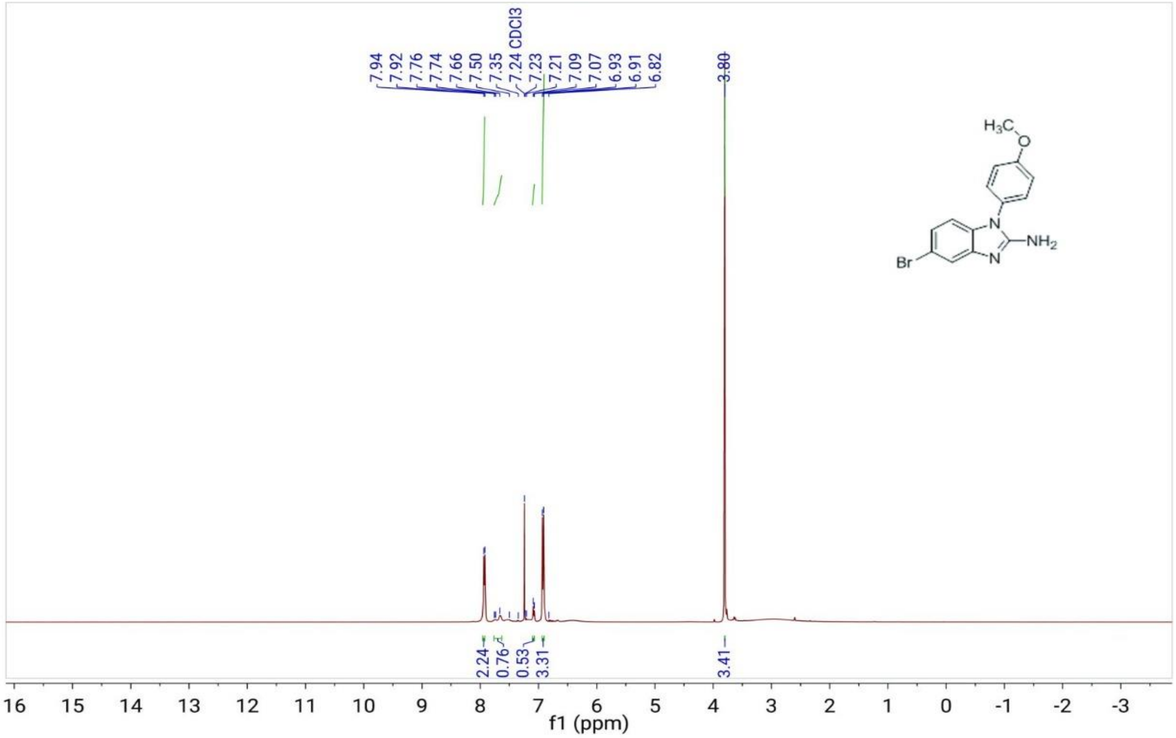

Figure S3. ^{1}H NMR (400 MHz, $CDCl_3$) of compound **1b**.

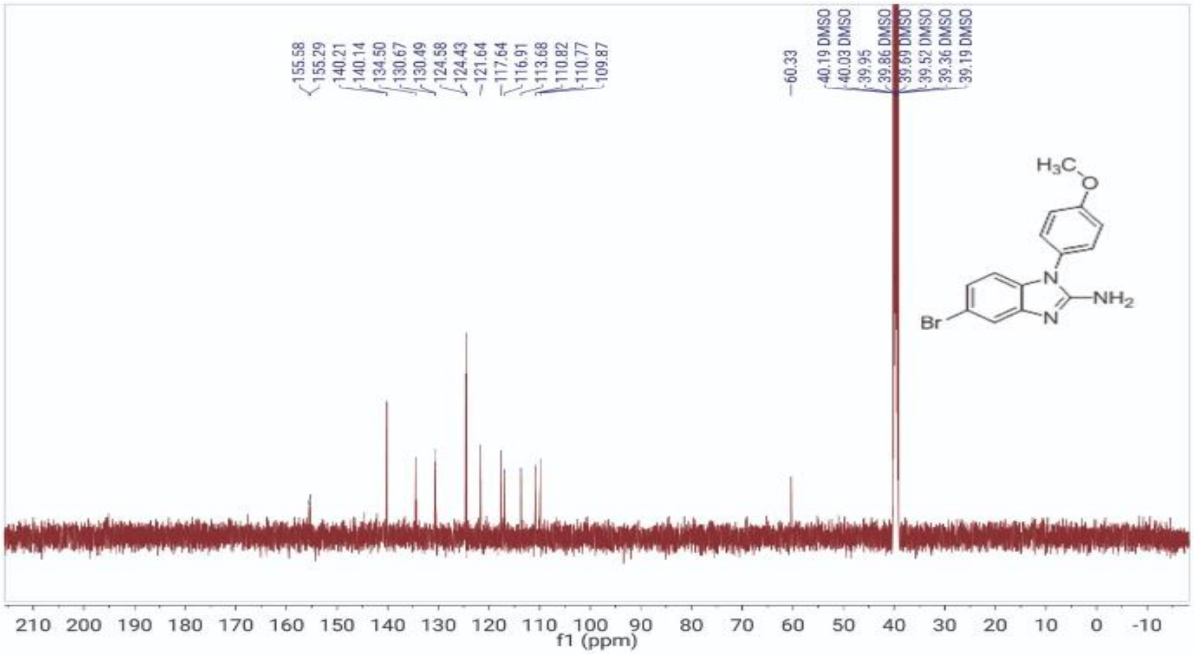

Figure S4. ^{13}C NMR (125 MHz, DMSO-d_6) of compound **1b**.

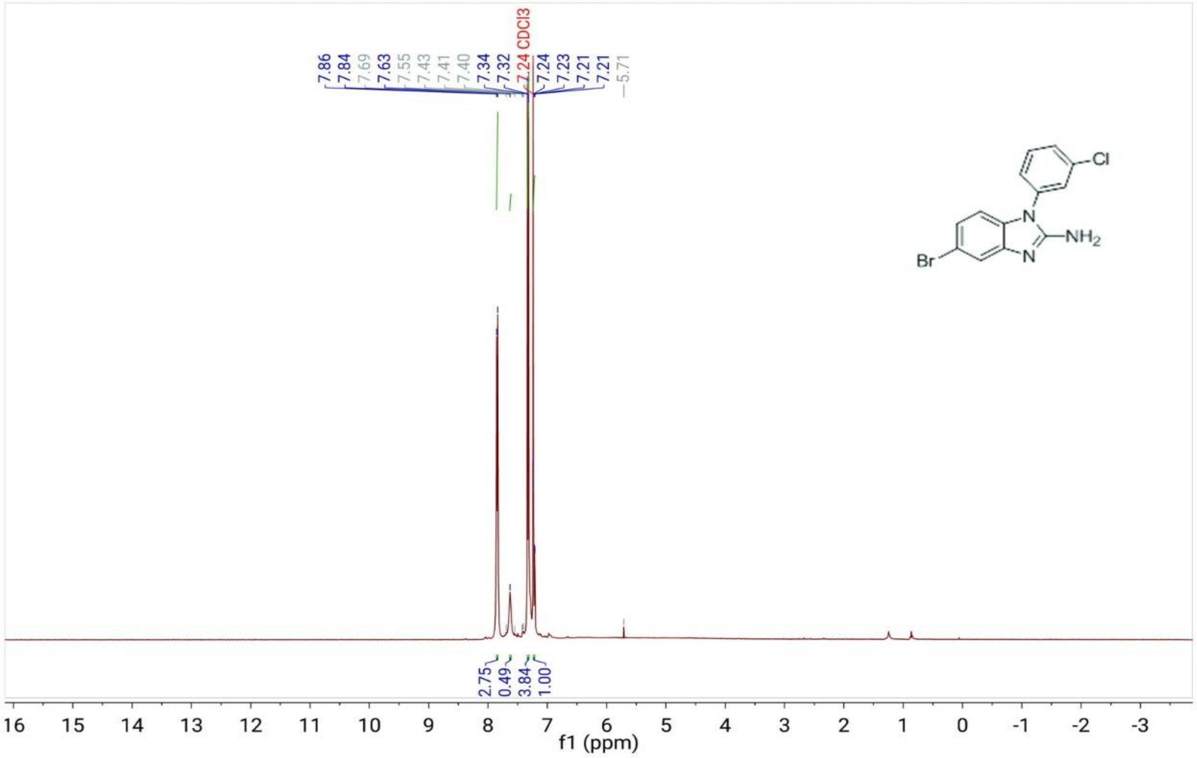

Figure S5. ^{1}H NMR (400 MHz, $CDCl_3$) of compound **1c**.

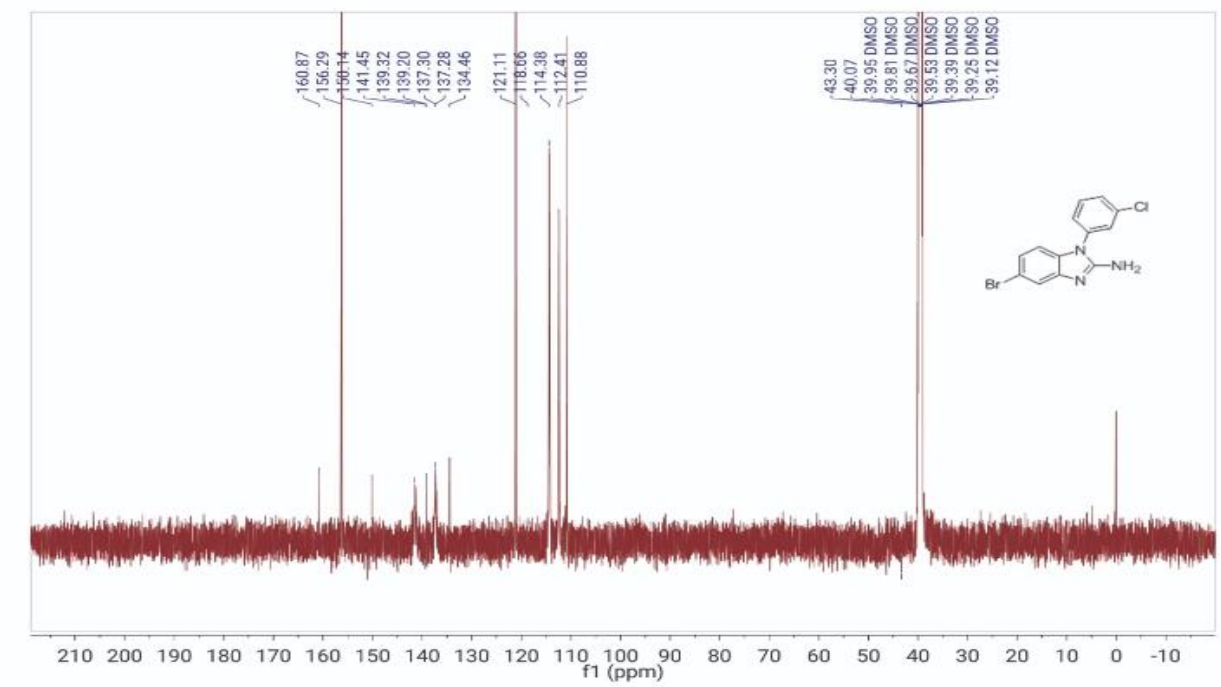

Figure S6. ^{13}C NMR (125 MHz, DMSO-d_6) of compound **1c**.

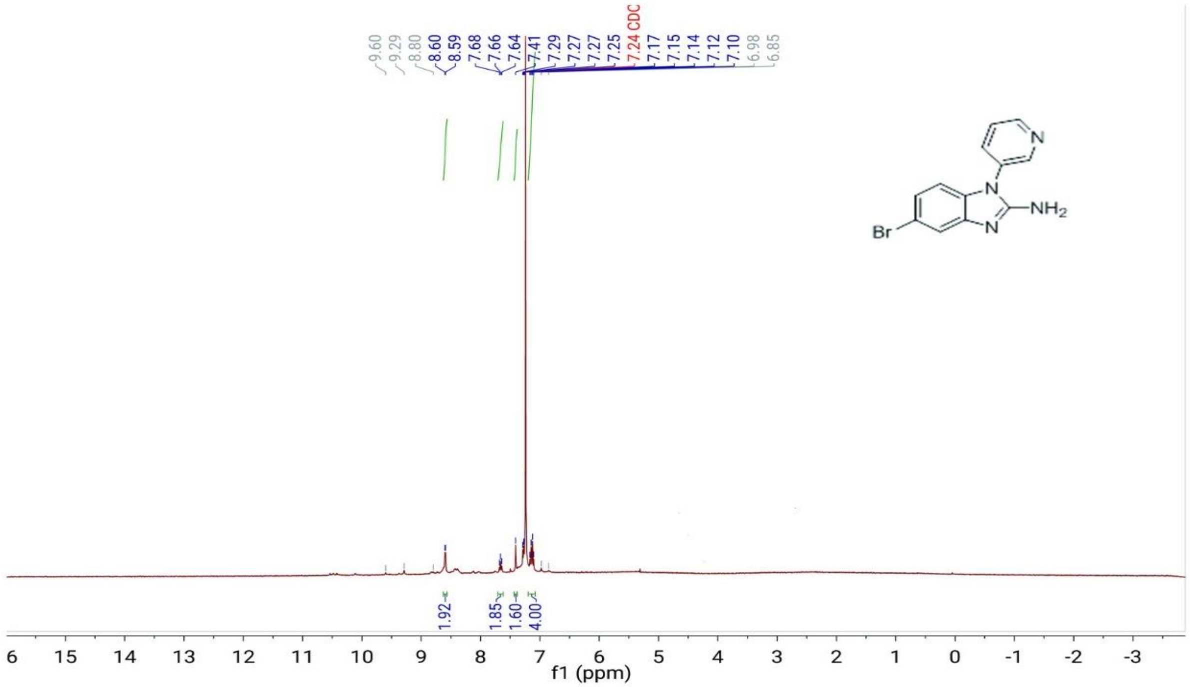

Figure S7. ^{1}H NMR (400 MHz, $CDCl_3$) of compound **1d**.

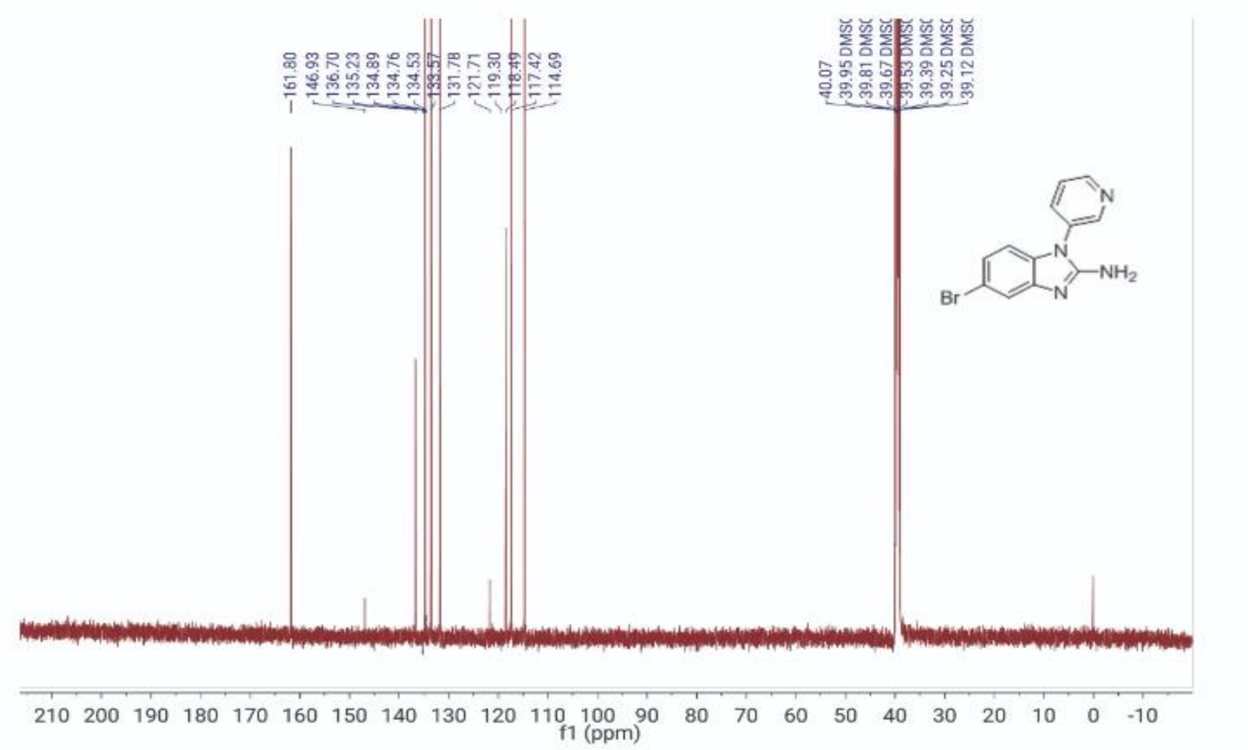

Figure S8. ^{13}C NMR (125 MHz, DMSO-d_6) of compound **1d**.

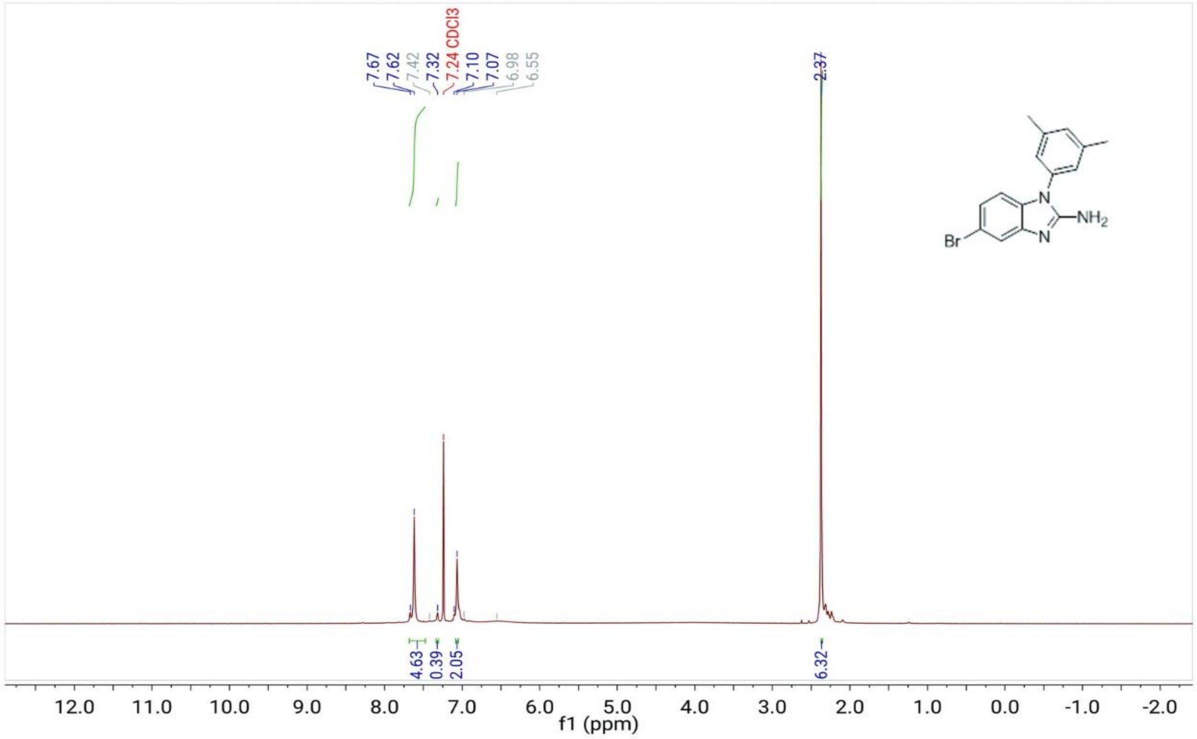

Figure S9. ^{1}H NMR (400 MHz, $CDCl_3$) of compound **1e**.

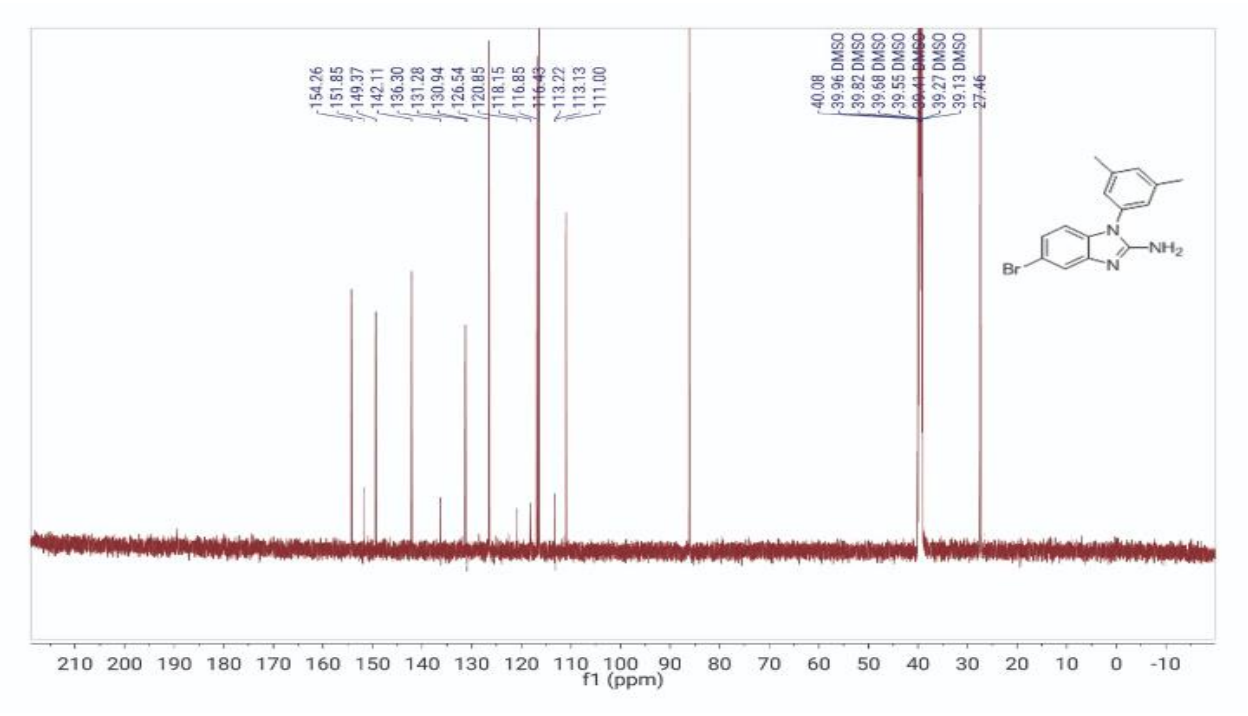

Figure S10. ^{13}C NMR (125 MHz, DMSO-d_6) of compound **1e**.

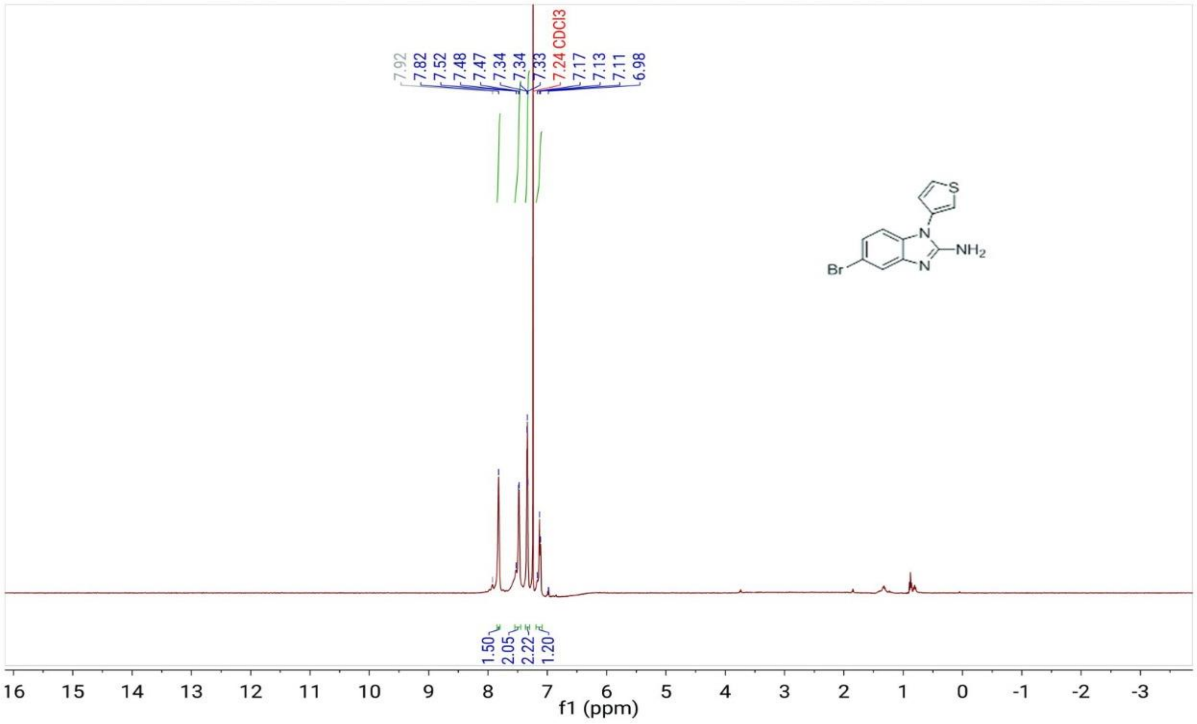

Figure S11. ^{1}H NMR (400 MHz, $CDCl_3$) of compound **1f**.

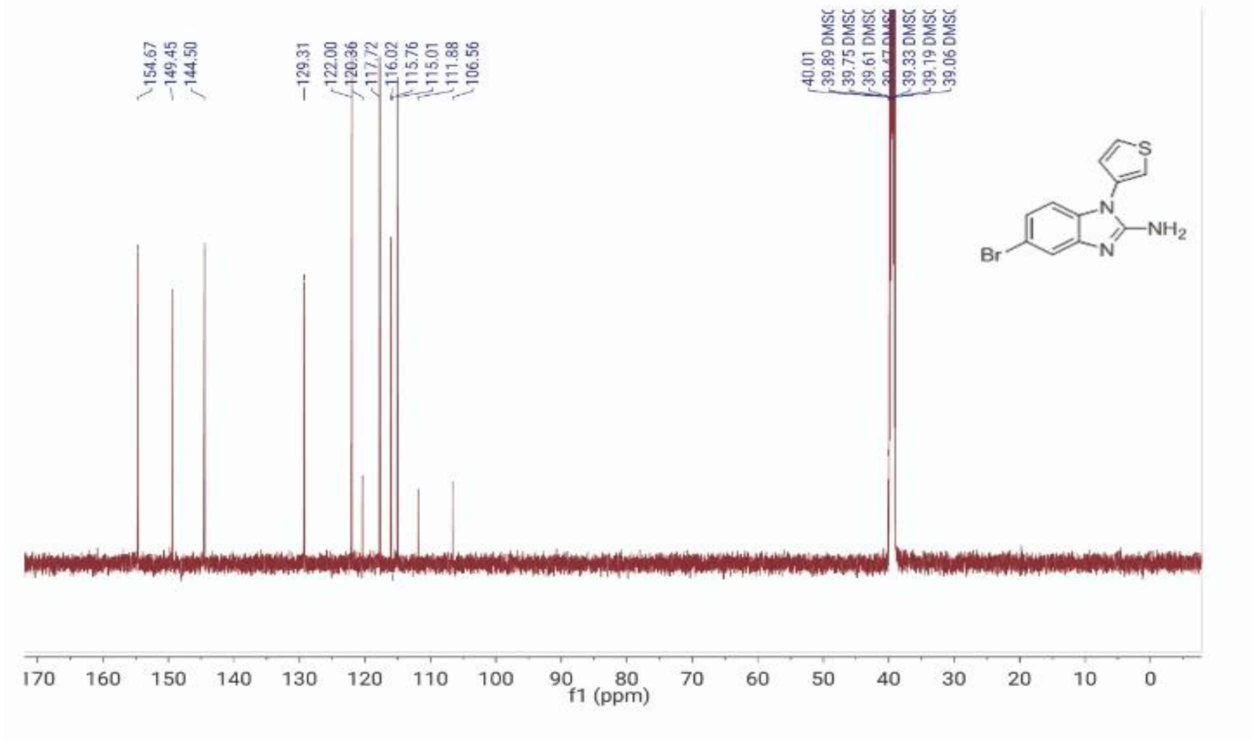

Figure S12. ^{13}C NMR (125 MHz, DMSO-d_6) of compound **1f**.

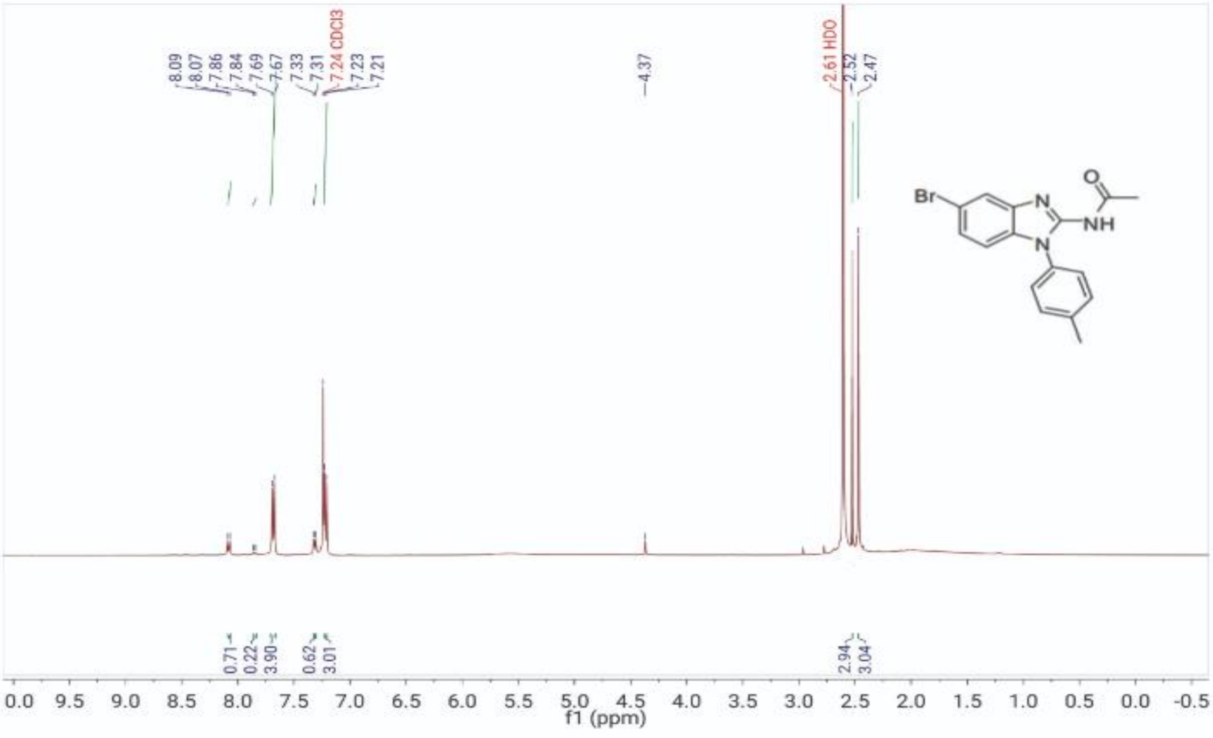

Figure S13. ^{1}H NMR (400 MHz, $CDCl_3$) of compound **2a**.

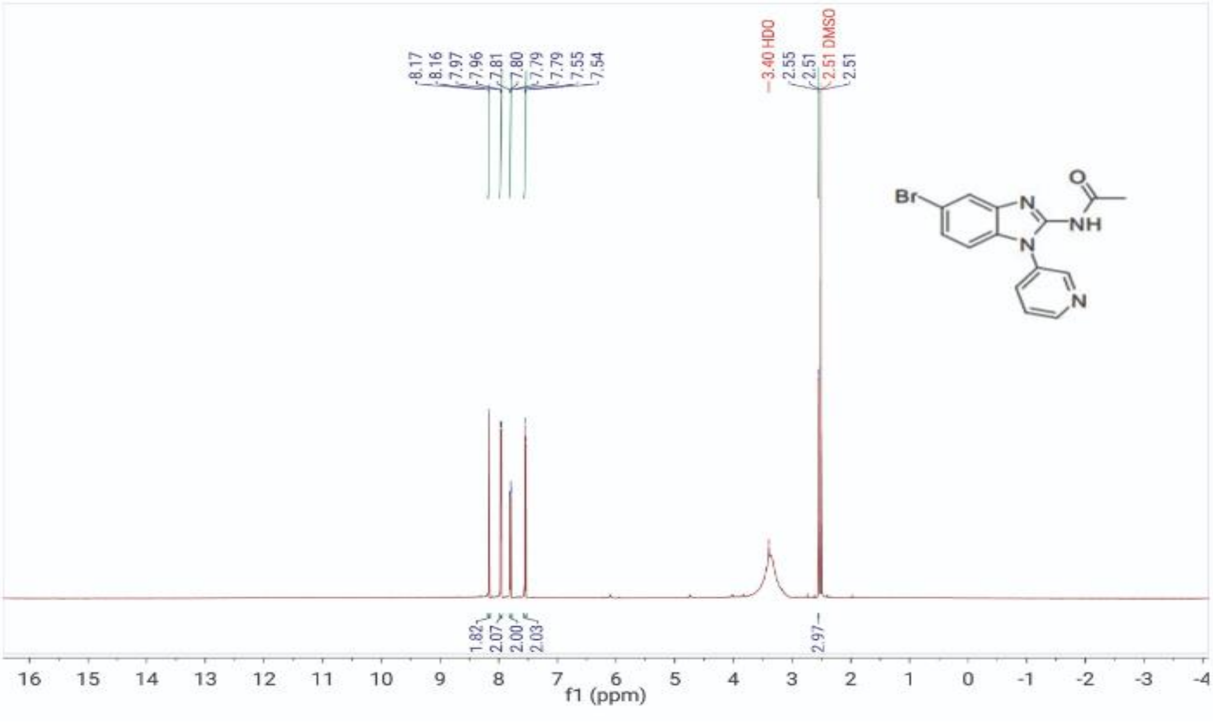

Figure S14. ^{1}H NMR (400 MHz, DMSO-d_6) of compound **2d**.

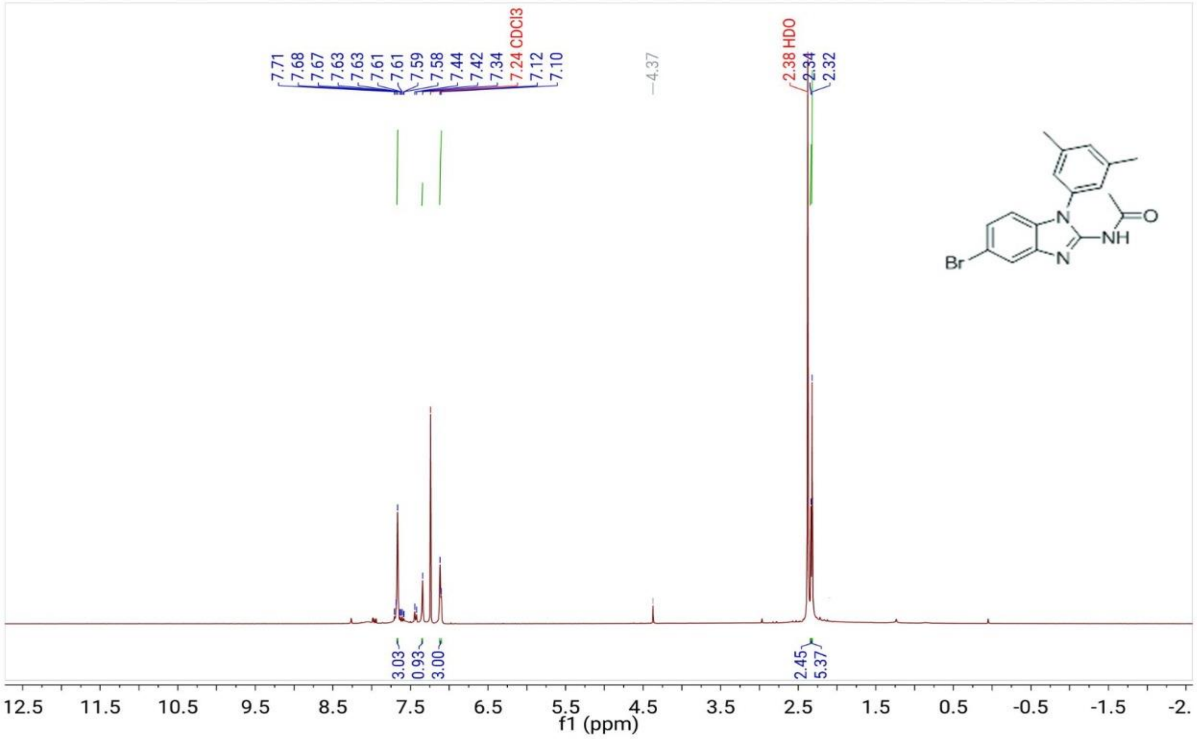

Figure S15. ^{1}H NMR (400 MHz, $CDCl_3$) of compound **2e**.

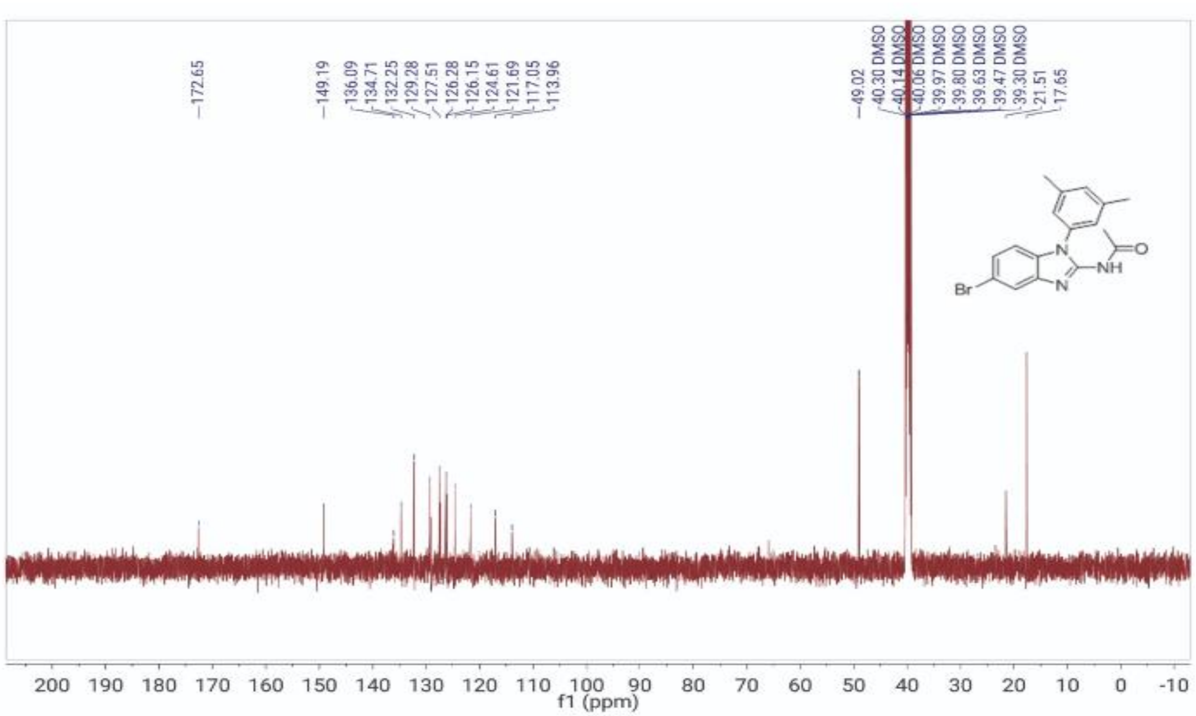

Figure S16. ^{13}C NMR (125 MHz, DMSO-d_6) of compound **2e**.

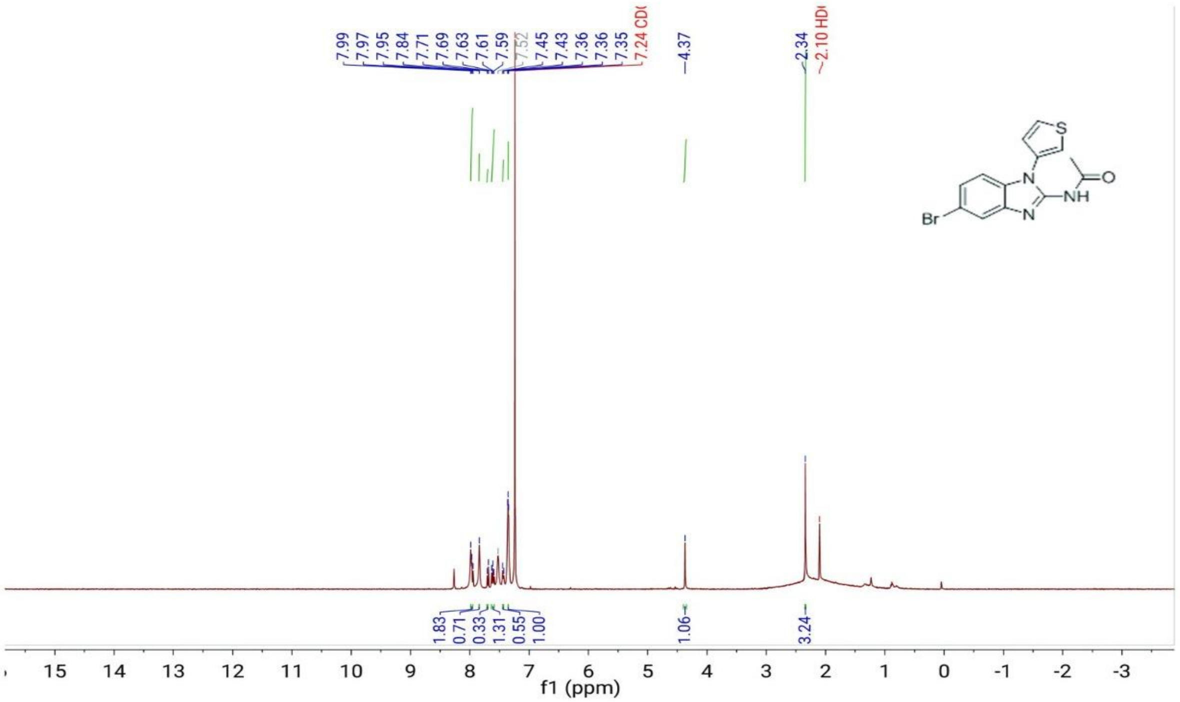

Figure S17. ^{1}H NMR (400 MHz, $CDCl_3$) of compound **2f**.

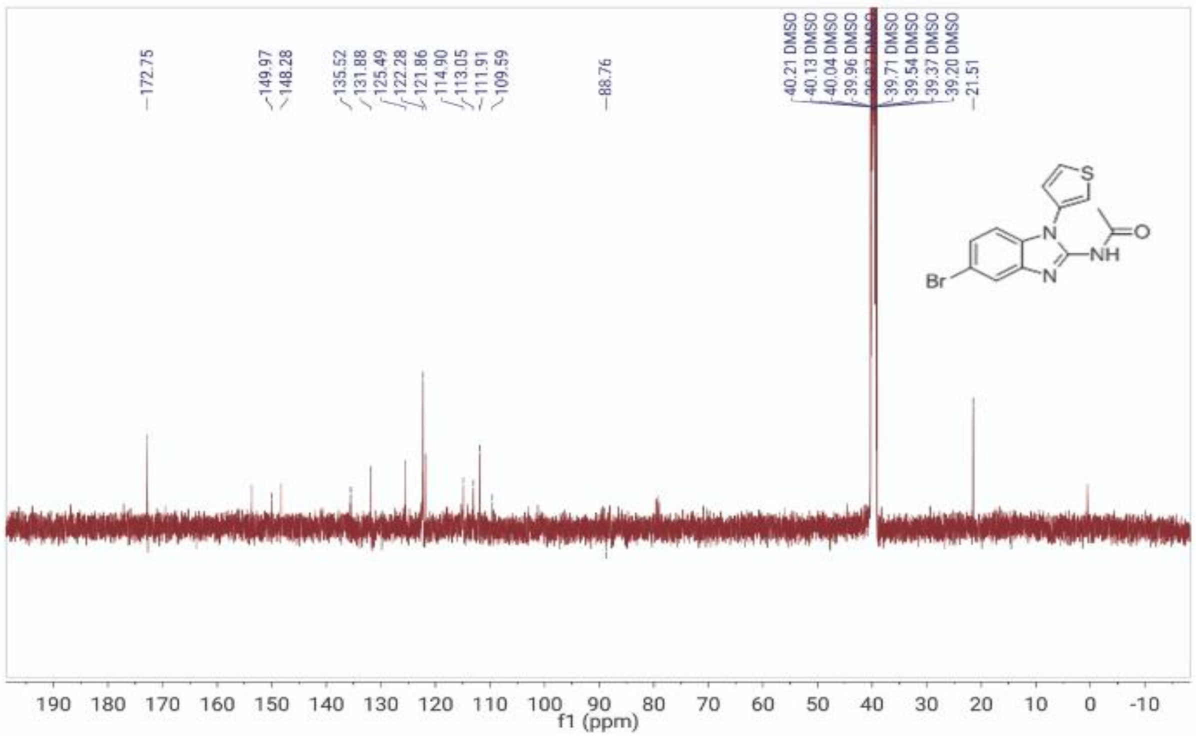

Figure S18. ^{13}C NMR (125 MHz, DMSO-d_6) of compound **24**.

Table S1. Polarizability (α_o, in au) and hyperpolarizability (β_o, in au) of compounds**1a-1f** and **2a-2f** at LC-BLYP, and ωB97XD.

Compounds	LC-BLYP		ωB97XD	
	α_o	β_o	α_o	β_o
**1a**	220	3.69×10^{-30}	227	4.40×10^{-30}
**1b**	208	5.63×10^{-30}	215	5.31×10^{-30}
**1c**	203	3.83×10^{-30}	210	3.53×10^{-30}
**1d**	183	3.89×10^{-30}	189	4.17×10^{-30}
**1e**	214	3.82×10^{-30}	221	3.70×10^{-30}
**1f**	182	3.98×10^{-30}	188	3.99×10^{-30}
**2a**	228	1.81×10^{-30}	235	1.93×10^{-30}
**2b**	232	3.82×10^{-30}	239	3.44×10^{-30}
**2c**	228	1.89×10^{-30}	236	1.90×10^{-30}
**2d**	208	1.80×10^{-30}	215	2.27×10^{-30}
**2e**	239	1.73×10^{-30}	246	1.95×10^{-30}
**2f**	208	1.98×10^{-30}	214	2.44×10^{-30}
